# Biologically meaningful genome interpretation models to address data underdetermination for the leaf and seed ionome prediction in *Arabidopsis thaliana*

**DOI:** 10.1038/s41598-024-63855-6

**Published:** 2024-06-08

**Authors:** Daniele Raimondi, Antoine Passemiers, Nora Verplaetse, Massimiliano Corso, Ángel Ferrero-Serrano, Nelson Nazzicari, Filippo Biscarini, Piero Fariselli, Yves Moreau

**Affiliations:** 1 ESAT-STADIUS, KU Leuven, 3001 Leuven, Belgium; 2 Université Paris-Saclay, INRAE, AgroParisTech, Institute Jean-Pierre Bourgin for Plant Sciences (IJPB), 78000 Versailles, France; 3 Department of Biology, Pennsylvania State University, University Park, PA 16802 USA; 4 CNR-IBBA, 20133 Milan, Italy; 5 CREA-ZA, 26900 Lodi, Italy; 6 Department of Medical Sciences, University of Torino, 10123 Turin, Italy

**Keywords:** *Arabidopsis thaliana*, Deep learning, Ionome prediction, Genomic prediction, Genome interpretation, Bioinformatics, Genomic analysis, Genetics, Agricultural genetics, Genomics, Plant genetics, Quantitative trait, Computer science

## Abstract

Genome interpretation (GI) encompasses the computational attempts to model the relationship between genotype and phenotype with the goal of understanding how the first leads to the second. While traditional approaches have focused on sub-problems such as predicting the effect of single nucleotide variants or finding genetic associations, recent advances in neural networks (NNs) have made it possible to develop end-to-end GI models that take genomic data as input and predict phenotypes as output. However, technical and modeling issues still need to be fixed for these models to be effective, including the widespread underdetermination of genomic datasets, making them unsuitable for training large, overfitting-prone, NNs. Here we propose novel GI models to address this issue, exploring the use of two types of transfer learning approaches and proposing a novel Biologically Meaningful Sparse NN layer specifically designed for end-to-end GI. Our models predict the leaf and seed ionome in *A.thaliana*, obtaining comparable results to our previous over-parameterized model while reducing the number of parameters by 8.8 folds. We also investigate how the effect of population stratification influences the evaluation of the performances, highlighting how it leads to (1) an instance of the Simpson’s Paradox, and (2) model generalization limitations.

## Introduction

Understanding genomes to the point of reliably modeling the relationship that leads from genotype to phenotype is one of the main goals of genetics^1^. This step is crucial to open new horizons for life sciences, including molecular biology, agricultural technology and precision medicine^2–4^. Computational attempts to model the genotype-phenotype relationship fall under the term Genome Interpretation (GI)^5–8^, Genomic Prediction^9,10^ or Genomic Selection^4^. Historically, the Bioinformatics approaches to investigate this relationship included linear mixed models^10–12^, Genome Wide Association Studies (GWAS)^13,14^, Polygenic Risk Scores (PRS)^15,16^, and various computational tools aiming at predicting the functional effect of Single Nucleotide Variants (SNVs) in humans^17,18^, animals^19^ and crops^20^.

The increasingly large amount of genomic data available nowadays is starting to make it possible to build advanced machine learning (ML) methods for GI. In particular, thanks to the flexibility of recent neural network (NN) libraries such as PyTorch^21^, it is now possible to develop *end-to-end* (e2e) GI models that directly take genomic data as input and predict the desired phenotypes as output, in a unique stream of computation. Notwithstanding the scientific opportunity to apply the latest NN methods to life sciences has been shown in many contexts^22,23^, among which the most striking example is surely AlphaFold^24^, in order to apply e2e NNs to GI, several technical and modeling issues need to be addressed^25^.

In our previous work, we proposed e2e NN methods for the Whole Exome Sequencing (WES)-based *in silico* risk prediction of Chrohn’s Disease (CD)^8,25^ and for the Whole Genome Sequencing (WGS)-based multitask prediction of 288 quantitative traits in the model plant *Arabidopsis thaliana* (AT)^5^, focusing on addressing the above-mentioned issues. For example, we proposed a compact yet informative feature encoding for WES/WGS data, and we tried to address the common *underdetermination* of genomic datasets by building models with an effective number of trainable parameters proportional to the dataset sample size, to limit the risk of overfitting and to ensure the robustness of the learned biological patterns^25^.

When predicting a single phenotype, this is relatively straightforward to achieve simply by considering as input only a limited set of phenotype-associated genes instead of the whole exome, resulting in a model with a number of parameters proportional to the sample size^25^. Unfortunately, this is generally not applicable when multiple phenotypes with possibly wildly different genetic causes need to be predicted, such as in the case of our model (called Galiana) for the multi-phenotypic prediction of 288 AT quantitative traits^5^. In this broader context, each of the 27655 AT genes could be involved in at least one of the considered phenotypes. Notwithstanding the efforts to limit the complexity of the Galiana model by using parameter sharing wherever possible, this design issue led to a model that was grossly over-sized with respect to the number of samples in the dataset (1.4M parameters,  1000 samples), and we relied on dropout and regularization to reduce the risk of overfitting^5^.

Despite the increasing production and collection of sequencing data, datasets underdetermination is still an ubiquitous issue when it comes to genomic data and GI. This is because sequencing costs are still not negligible, and sample collection is a tricky procedure. Sequencing technologies measure a considerable number of variables $m \in M$ for each sample $n \in N$, thus leading to an unfavorable $|N|\ll |M|$ situation, since it might lead to severe overfitting.

In this work, we addressed the issue of performing GI inference on underdetermined WES/WGS datasets, comparing our previous over-parameterized Galiana model with alternative NN architectural solutions that drastically reduce the model complexity, while still allowing non-linear inference. In particular, we explore the use of two types of transfer learning (TL) approaches and we propose a novel Biologically Meaningful Sparse NN (BMSNN) layer specifically designed for e2e GI.

We apply these GI NN models to the prediction of the leaf and seed ionome in AT, using the largest and most recent experimental determination of the concentration of 18 elements in the seed and leaf tissues of the largest available collection of AT samples, representative of the entire genetic variability in this species^26^. The plant ionome corresponds to its macro (e.g. Ca, K, Na, Mg) and trace metals (e.g. Fe, Mn, Zn, Se) and is regulated by multiple and complex genetic and environmental factors^26^, such as nutrient deficiencies and other stresses. Therefore, it can be used to characterize the physiological status of the plant^27^ by identifying biomarkers that reflect stress conditions^28^. Combining the plant ionome with genetic information can be used to identify genes that regulate micro and macronutrients homeostasis^27,29^, and studying the dynamics of mineral elements and their genetic regulation is useful for biofortification and improving crop nutritional values^30^.

We show that while our previous NN GI architecture for multi-phenotypic prediction of AT quantitative traits (Galiana) can be adapted to the ionome prediction, comparable results can be obtained while drastically reducing the number of trainable parameters by sparsifying the NN architecture with our BMSNN layer, which is specifically designed for GI and has the peculiarity that both input and output neurons represent biological entities, such as genes or pathways. Only the weights mirroring existing connections between these entities (i.e. edges in a gene-gene interaction network, or a gene-pathway hierarchy) are allowed, thereby exploiting the intrinsic small-worldness of biological networks to reduce the model complexity, and therefore also the risk of overfitting.

Additionally, we analyse the effect of population stratification on the predictions, showing the challenges of measuring model performance in heterogeneous populations^31^. In such cases, accounting for stratification in the cross-validation procedure and the calculation of performance is essential to avoid producing incorrect results.

## Methods

### Dataset

#### Ionome data

In^26^, the authors experimentally quantified the concentrations of 18 mineral elements in the leaf and seed tissues of AT samples from the 1001 Genomes Project^32^. The elements measured are Se82, Mo98, Mn55, Cd114, Sr88, Mg25, K39, Rb85, Cu65, Ca43, P31, S34, Li7, As75, Na23, Fe57, Zn66 and Co59. The full data are available at https://ffionexplorer.nottingham.ac.uk/ionmap/. Since the mean and variance of these values are element-specific^26^, we standardized the concentrations of each element individually to a Gaussian distribution ${\mathcal {N}}(\mu =0, \sigma ^2=1)$. Ionome elements autocorrelations are shown in Suppl. Fig. S51.

#### *A. thaliana* WGS data

From the 1001 Genomes Project database we collected 1135 AT WGS samples and matched them with the corresponding ionomic profile from^26^. We obtained a dataset containing 998 AT WGS samples belonging to 46 countries labeled with 18+18 element concentrations from leaf and seed. Similarly to^5^, we annotated these VCF files with Annovar, but, due to the scarcity of functional annotations for AT genomes, we could only retrieve information regarding the variant types and the genomic regions on which they are mapped. Each variant in the AT WGS is thus assigned to one of the 17 following types: (exonic) nonsynonymous, (exonic) non-frameshift insertion, (exonic) non-frameshift deletion, (exonic) stoploss, (exonic) frameshift insertion, (exonic) frameshift deletion, UTR3, UTR5, exonic ncRNA, intronic ncRNA, upstream, downstream, intergenic, intronic, splicing, ncRNA splicing, exonic stopgain.

### Transforming AT WGS data into NN-understandable input tensors

Following our previous work on the development of NN models for GI^5,25^, we transformed the input WGS data into ML-ready vectors by converting the Annovar annotations into a *gene-centric* feature encoding, where each of the 27655 AT genes is represented by a 17-dimensional histogram counting the number of occurrences of each of the 17 types of variants on it. The genome of each AT sample is thus described by a (17, 27655) vector. This encoding conceptually provides a mutation type-specific quantification of the *gene mutational damage* of each AT gene, and provides a coarse-grained, yet convenient way to represent AT WGS data into ML-understandable vectors. As a last processing step, to avoid numerical problems in the NN, we standardized the integer counts of the 17 types of variants on all genes and all samples in the dataset^5^. This procedure is illustrated in Suppl. Fig. S55.

### Genome interpretation solutions to address data underdetermination for the ionome prediction

We present three novel e2e NN GI approaches that address the ubiquitous underdetermination of genomic datasets, and apply them to the prediction of the seed and leaf ionome of AT.

**Figure 1:**
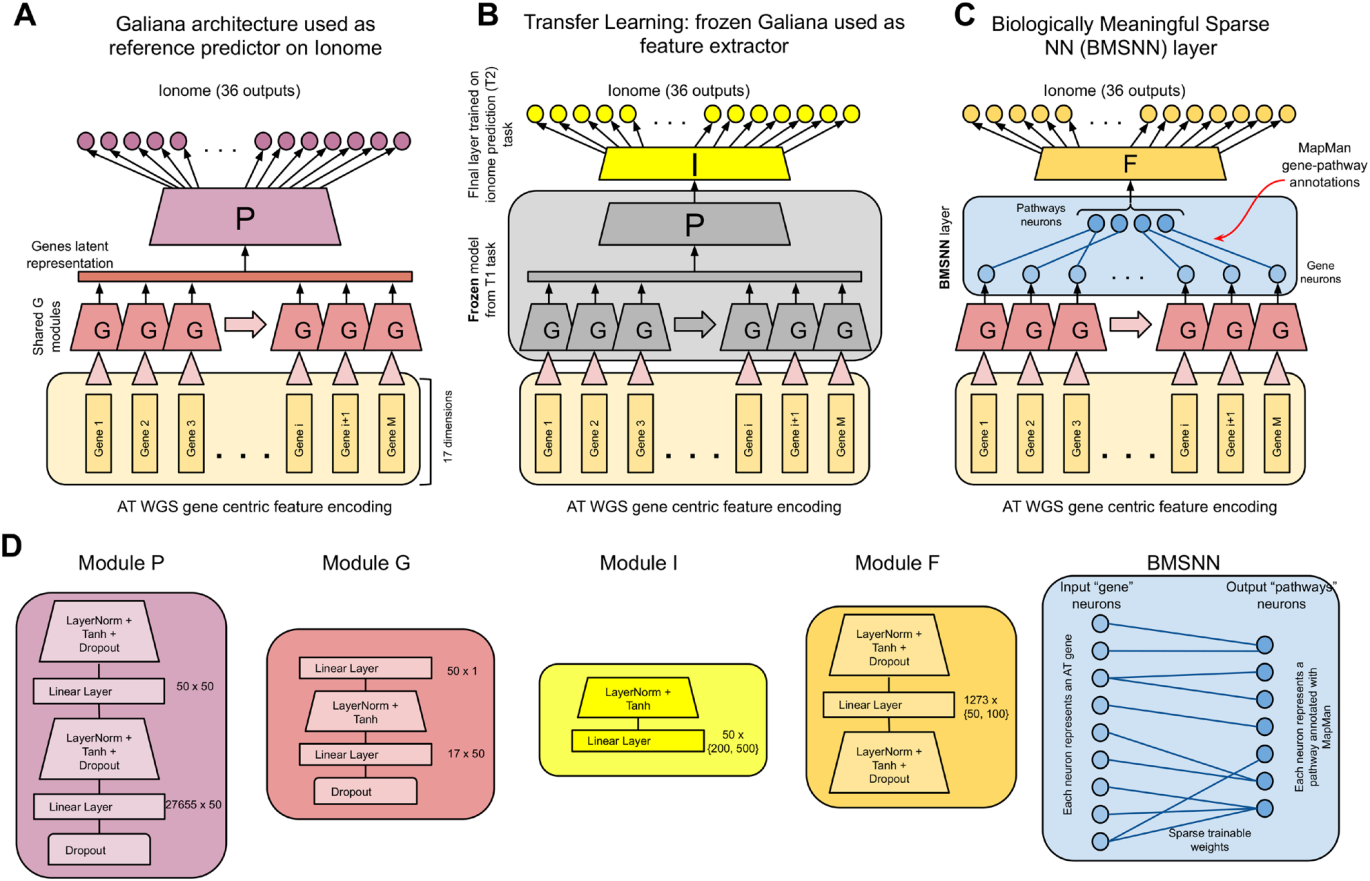
Overview figure showing the NN GI models used in this paper (**A–C**). (**A**) The Galiana architecture, adapted for the ionome prediction (36 output neurons). (*B*) The frozen Galiana architecture trained on the 288 AT phenotypes from^5^ (grey part) as unsupervised feature extractor. Only the *I* module [see details in (**D**)] is optimized for ionome prediction. (**C**) The Biologically Meaningful Sparse NN layer (BMSNN) we propose to reduce the model complexity by incorporating biological networks into our model. The input neurons of the BMSNN layer are the 27,655 genes, while the 1273 outputs represent biological pathways. Only the weights corresponding to knowledge-based edges connecting genes and pathways are allowed, thus making it sparse (27,655 weights). (**D**) The details of the modules used in these NNs.

#### Adapting Galiana architecture to the ionome prediction

As a baseline, we adapted our previously published Galiana model^5^ for the multi-task regression of the 18 elements measured in leaves and seeds (36 tasks in total). Given the gene-centric feature encoding representing each AT sample as a (27,655, 17) input tensor, Galiana first applies a shared *G* module on the 17 dimensional representation describing each gene, using two layers to compress the 17 dimensions to 1 single value describing the gene mutational load, obtaining a (27,655, 1) vector (see Fig. 1A for an illustration). A multi-task *P* module is applied on them, leading to the 36 real-valued regression tasks.

Notwithstanding the attempts to limit the number of trainable weights, for example by using a shared module *G* to process the input of all genes (see Fig. 1A), and by summarizing each gene into a single latent value, Galiana still has 1.4M trainable parameters, due to the fact that the module *P* must necessarily be a fully connected layer to integrate the neuron activations describing the mutational load of the 27,655 AT genes. We use the Galiana model as a reference in terms of the predictions performances that we can obtain on the ionome prediction, comparing it with the smaller models we propose.

#### Transfer learning experiments

Transfer learning (TL) exists in many forms, depending to the specific problems that must be addressed. See^33^ for a comprehensive review. Generally speaking, TL encompasses ML efforts focusing on training a model ${\mathcal {M}}$ to solve a certain task $T_1$ and then using the patterns it learned to solve a different but related task $T_2$. A possible reason for TL could be that not enough data is available to train ${\mathcal {M}}$ from scratch on $T_2$, while a larger dataset is available on the related $T_1$ task.

Here we focus on the situation in which the feature space of the two tasks is the same (the AT genomes from^32^), but where the label space is different. In particular, we used the 288 phenotypes from Arapheno^34^ as prediction labels for the task $T_1$ and the 36 element concentrations as labels for $T_2$. This is generally called *Inductive TL*, since the goal is to exploit the inductive bias of $T_1$ to help the prediction of $T_2$^35^. Within these settings, we tested the following two TL approaches.

#### Frozen $T_1$ model as feature extractor

In this scenario, we trained the Galiana architecture to predict the 288 phenotypes ($T_1$), as described in the original paper (see Fig. 1A). We then froze the weights, not allowing any further optimization on them, and removed the last layer (which is indeed specific for $T_1$) replacing it with a new trainable module *I* (see Fig. 1B) consisting in $H=\{200,500\}$ hidden neurons with LayerNorm and Tanh activation going into a final layer with 36 neurons predicting the ionome ($T_2$). We thus used the *body* of the Galiana architecture (modules G, P) as *unsupervised feature extractors*, assuming that the latent encoding of the AT genomes they learned for $T_1$ could also be informative for $T_2$. We trained only 17,836 out of the 44,536 weights of the *I* module on the ionome dataset, using the RMSprop optimizer with *L*2 regularization ($10^{-6}$) and 0.01 learning rate. We ran this experiment with $H=\{200,500\}$ neurons, and we called these two models “frozen200” and “frozen500” in the rest of the paper. The training is performed with a specific cross-validation to avoid leakage from task $T_1$ to $T_2$. The procedure is described in “Implementation and validation”.

#### Fine tuning of pre-trained $T_1$ model

In this second TL approach, we first fully trained Galiana architecture on $T_1$, and then we *fine tuned* it for $E=\{30,50\}$ epochs on the ionome dataset ($T_2$). In this case, training on $T_1$ data acts as an informed *weight initialization* that could improve the convergence of the training on $T_2$. We refer to these models trained for 30 and 50 epochs as “transf30” and “transf50” from now on. The training is performed with the cross-validation described in “Implementation and validation”.

#### Biologically meaningful sparse layers for genome interpretation

TL can be a way to limit the model complexity and overcome the scarcity of data for a certain target task $T_2$, but it requires sufficient data to be available for a related task $T_1$ with a useful inductive bias towards $T_1$ prediction. Unfortunately, due to the aforementioned underdetermination of genomics dataset, this scenario is not likely to be very common. We thus also propose a different solution in the form of a GI-specific NN layer that exploits the small-world properties of the biological networks underlying the genotype-phenotype relationship to sparsify the NN trainable parameters in a biologically meaningful manner (see Fig. 1C). We call this layer Biologically Meaningful Sparse NN (BMSNN). Its main peculiarity is that both input and output neurons represent biological entities (i.e. genes or pathways). Only the parameters mirroring existing connections between these entities (i.e. edges in a gene-gene interaction network, or a gene-pathway hierarchy) are allowed, thus resulting in a knowledge-based sparsification of the otherwise dense feed-forward NN layer which results in a significantly lower number of parameters. In Fig. 1C we show how we used this layer in the Galiana architecture to replace the dense *P* module, obtaining a 88% reduction of the number of parameters. Following the original architecture, the input neurons in the BMSNN layer represent the 27,655 AT genes, while the 1273 output nodes represent the AT metabolic pathways and biological processed downloaded from MapMan^36^. 72% of the 27,655 AT genes considered in this study were associated with at least one MapMan entry. The remaining unannotated genes were connected to a *dummy* pathway/neuron we added to avoid discarding the information coming from these genes.

### Implementation and validation

#### Implementation

All proposed models have been implemented with PyTorch^21^. The models have been trained for 50 epochs (except where specified otherwise) with the Mean Squared Error (MSE) loss and batch size of 10. Similarly to^5^, the learning rate was set to $10^{-2}$ with a reduction of 1/3 every 3 consecutive epochs without loss decrease. We used L2 regularization with $\lambda = 10^{-6}$ for the Galiana model and $\lambda = 10^{-4}$ for the BMSNN. We also performed a nested cross-validation with hyper parameter optimization using Optuna^37^: see Suppl. Mat. Section S5 for more details on the optimization procedure.

#### Validation

We collected the predictions of the Galiana, BMSNN50/100 and GPSNN models with a fivefold *random* cross-validation, meaning that the samples were randomly assigned to folds. We evaluated the predictions by computing the Pearson correlation between the predicted and observed ionome concentrations within each of the seven countries with more than 50 samples available (Sweden, Spain, USA, Germany, Italy, UK and Russia). We then averaged these *intra-country correlations* for each element to obtain the final scores (see “Results” for more details). Additionally, we also ran a cross-validation *stratified by country*, meaning that in each iteration the samples from a nation ${\mathcal {N}}$ could be present either in the training and in the testing (see “Discussion” for more details).

#### Transfer learning validation

We computed the predictions of the TL-based models (transf50/30epochs and frozen200/500) by using a specific fivefold cross-validation procedure to avoid any unwanted information leakage from the $T_1$ task to $T_2$. In each cross-validation iteration, we use 80% of the samples for training and 20% for the test set. Within each iteration, we first train our old Galiana model for the prediction of the 288 Arapheno^34^ phenotypes (Task $T_1$) associated with the training set samples. In the case of the frozen200/500 models, we then freeze these trained weights, and we use this trained model as an unsupervised feature extractor, training *only* the TL model around it on the ionome data (TL Task $T_2$). In the case of the transf50/30epochs, we use these weights trained on the transf50/30epochs Task $T_1$ as initializations for the full model trained to solve the Task $T_2$. This CV procedure prevents data leakage, since the samples in the test set are never seen before by any of the trained models, regardless of the task ($T_1$ or $T_2$). We used the same hyper parameters used in^5^ to train the $T_1$ predictor.

#### GPSNN validation

As baseline predictor, we used the GPSNN model which takes as input only the GPS coordinates of the geographic origin of each AT sample, therefore modeling the kind of predictions that can be obtained by a pure geographical model that does not consider genetics aspects. We transformed the GPS (*lat*, *lon*) coordinates into radians and then we built a three dimensional feature vector $(\sin (lat), \sin (lon), \cos (lon))$, mapping them on the unit sphere.

## Results

### GI models to predict leaf and seed ionome while addressing the underdetermination of AT genomic data

In this paper, we explore three e2e Neural Networks (NN) Genome Interpretation (GI) architectures to address the ubiquitous underdetermination of genomic datasets, and we apply them to the prediction of the concentrations of mineral elements (collectively called ionome) in seed and leaf tissues of *A. thaliana* (AT).

Even though we used the largest genomic dataset available for AT^32^ ($N=998$ samples), coupled with an extremely compact way to encode sequencing data into NN-ready feature vectors, each sample is represented by $M=27,655$ vectors summarizing the mutational load carried by each AT gene (see “Transforming AT WGS data into NN-understandable input tensors”), resulting in a $N\ll M$ situation. We therefore focus on GI models capable of obtaining competitive performance while minimizing the number of trainable parameters.

**Figure 2:**
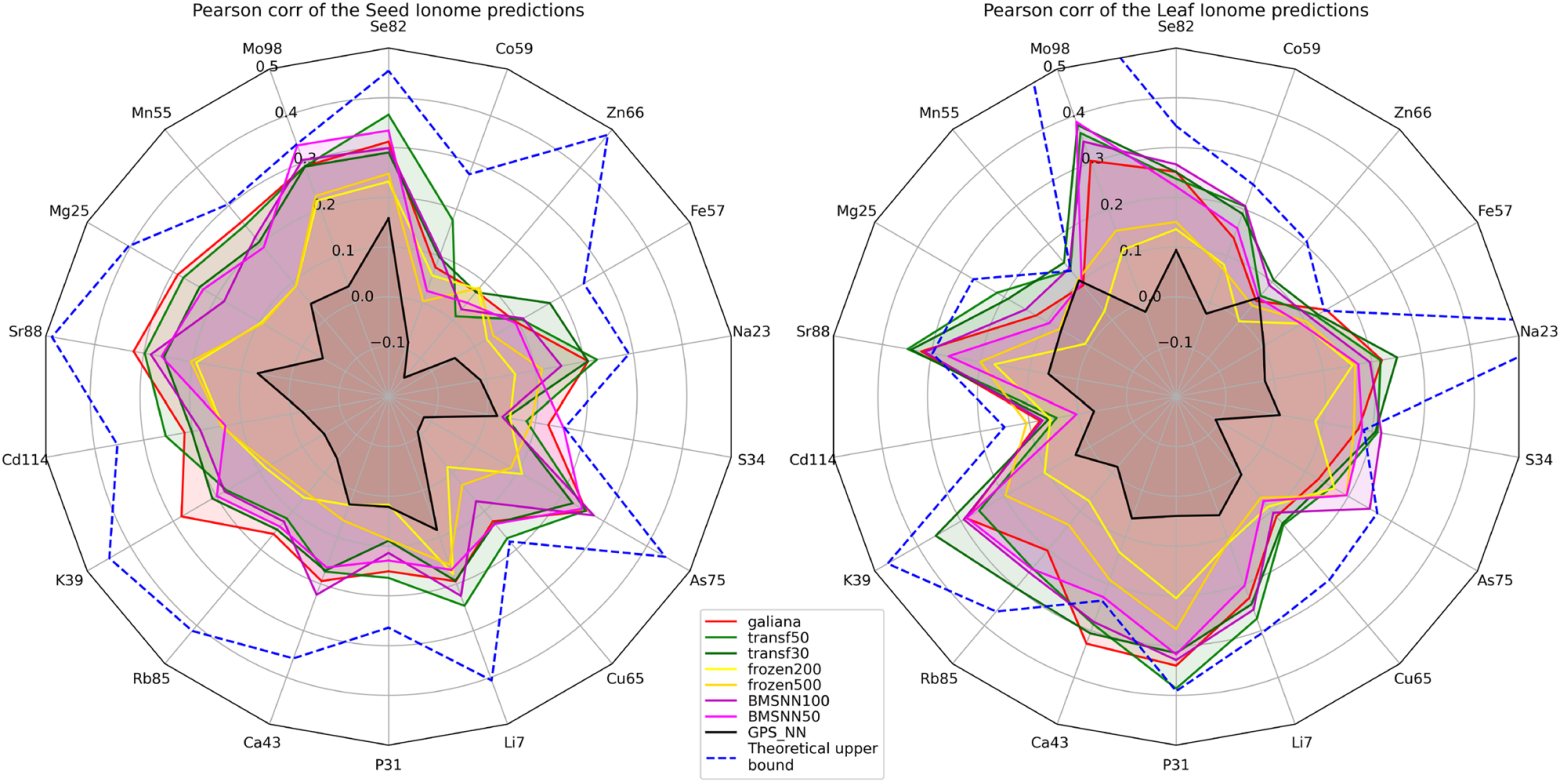
(**A**) Radar plots showing the Pearson correlation between the models predictions (colored lines) and the observed ionome concentrations on the seed (left plot) and leaf (right plot) tissues in AT. Each line represents a different model (see “Methods”) and indicates each minearl element intra-country correlations averaged over the 7 countries with more than 50 annotated AT samples. (**B**) Prediction performances expressed as a percentage of the theoretical Pearson upper bound due to experimental uncertainty.

#### Validation procedure

The AT samples with ionome concentrations measured by^26^ have been gathered from 46 countries (see the number of samples per country in Suppl. File S1). We trained and tested our model with a random fivefold cross-validation (CV) (see “Methods” ”Implementation and validation”). To evaluate the performance of our models, we selected the 7 countries with more than 50 annotated AT samples, which are Sweden (243 samples), Spain (180), USA (123), Germany (118), Italy (73), UK (69) and Russia (60). We then computed the Pearson correlation between the predicted and observed concentrations of each ionome element within each country. We averaged these intra-country correlations to obtain the average Pearson correlation between predicted and observed values for each of the 18 ionome elements. These results are shown with radar plots in Fig. 2.

The black line represents a baseline predictor that only models geographic aspects, ignoring the genetics of AT. It receives as inputs the GPS coordinates of each AT sample (see “Methods” “Implementation and validation”) and we use it to put the performance of our genetics-based methods in perspective.

#### Galiana and fine tuning TL

The red line shows the performance of our Galiana method for multi-phenotype prediction on AT samples^5^ after being adapted to ionome prediction. The two green lines (“transf30”, “transf50”) show the performance of the Transfer Learning (TL) fine-tuning approach described in ''Transfer learning experiments''. In this scenario, within each CV iteration, we pre-trained the Galiana architecture for the prediction of the 288 phenotypes from Arapheno^34^ (task $T_1$), and we then fine-tuned the model by retraining it for 30 and 50 epochs, respectively on the ionome dataset (task $T_2$). The intuition behind this TL approach is that the pre-training on the 288 phenotypes of task $T_1$ could act as an *informed initialization*, if $T_1$ carries a useful inductive bias towards the prediction of $T_2$^35^. On the seed ionome, this fine-tuning TL approach performs similarly to the standard Galiana architecture, providing some improvement on the Se82, Co59, Fe57, Cu65 and Li7 elements. On the leaf ionome it improves the predictions on elements like Mo98, Co59, Na23, K39, Rb85, P31 and Mg25.

#### Frozen TL feature extractor

The yellow lines show the performance of the other TL approach we tried, which is described in “Transfer learning experiments”. In this scenario, in each CV iteration we trained the Galiana architecture on the task $T_1$ (288 phenotypes from Arapheno), and then we *froze* the learned parameters, not allowing any further optimization on them. We then plugged a novel final layer on top of this trained NN (the module *I* in Fig. 1B) and we then trained only 17,836 parameters on the ionome prediction task $T_2$ (instead of the 1,443,747 of the entire Galiana model). This TL approach uses the frozen NN as an unsupervised feature extractor, and thus has the advantage of using 98,8% fewer weights than Galiana, but as shown in Fig. 2, it also performs significantly less than other approaches.

#### Biologically meaningful sparse NN layer (BMSNN)

The purple and magenta lines in Fig. 2 show the performance of the last NN GI approach we propose to mitigate the underdetermination of genomics dataset used for GI. In this case, we propose a novel Biologically Meaningful Sparse NN layer (BMSNN), which is shown in Fig. 1C, D. With this layer in our architecture, after the latent gene representations produced by the shared module *G* are pooled together, we are no longer forced to use a fully connected *P* module, as we did in our original Galiana architecture (see Fig. 1A). Instead, we exploit the natural sparsity of the biological networks underlying the genotype-phenotype relationship to build a sparse layer in which the input neurons represent the 27655 AT genes, and the output neurons represent the 1273 pathways from MapMan^36^. In this BMSNN layer, only the edges corresponding to known gene-pathway associations are allowed, thus providing a biologically meaningful way to reduce the total number of parameters in our GI model (see Fig. 1C, D). In the original Galiana architecture^5^, the module *P*, which is a fully-connected feed-forward layer that merges the the latent representation of all the AT genes, is where the vast majority of the trainable parameters (and thus the model complexity) is located, with 1385252 weights. With the BMSNN model we propose in Fig. 1C, we sparsified the main trunk of the NN, using 91306 or 154956 weights instead (93-88% reduction). Most importantly, this reduction in model complexity does not translate in a striking loss in performance (see Fig. 2), making our BMSNN approach viable for the development of future GI NN models.

**Figure 3:**
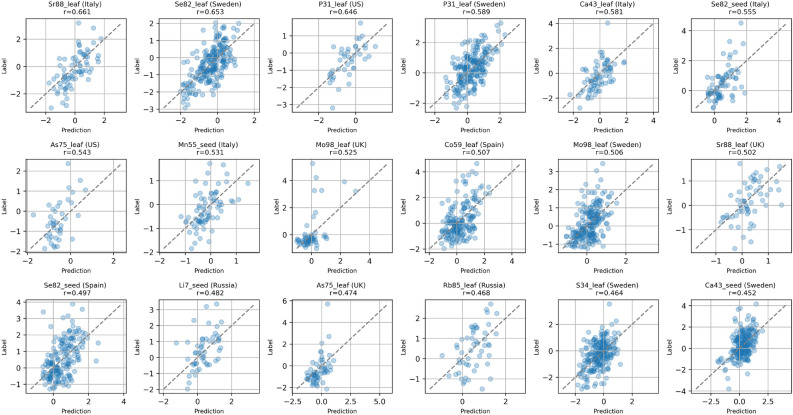
Scatter plots showing the 15 best predicted intra-country ionome elements with the BMSNN100 model.

### Contextualizing the predictions with the theoretical upper bound for Pearson correlation

The low Pearson correlation suggests that ionome prediction is a complex task, possibly because (1) it is characterized by high phenotypic plasticity and (2) trace and macro element accumulation and transport are driven by multigenic traits^38,39^. Nonetheless, when the goal is to perform a regression, the maximum achievable Pearson score between the predictions and the labels is not necessarily 1. Instead, a theoretical upper bound for Pearson scores can be derived^40^ from the relationship between the observations’ (i.e. the training labels) experimental uncertainty and the distribution of these observation across the entire dataset.

The concentrations of the 18 mineral elements in leaf and seed AT tissues were experimentally determined with six replicates^26^. For each element in each sample, we computed the variance between these replicates to estimate the experimental uncertainty, and we then followed the procedure described in^40^ to compute the upper bound of the Pearson correlation. For each ionome element, the dashed blue line in Fig. 2 shows this bound. To give a better perspective, Suppl. Fig. S1 shows the prediction performance as a percentage of the maximum theoretical Pearson correlation achievable on the current dataset. Fig. 2 showed that, even though the actual correlations are low, for some elements (i.e. Mo98, S34, Cu65 in seed and Fe57, As75, P31, in leaf), the models predictions are actually very close to the theoretical limit.

For other elements (i.e. Mn55, Sr88, Ca43, S34 in leaf), some models exceed this limit. This is likely due to two main aspects. First, the closer our model gets to the bound, the more it is likely to cross it by sheer chance due to random fluctuations, and indeed most of these cases occur at very low Pearson correlations. Second, since the bound is based on the estimation of the experimental uncertainty from replicates, the relatively low number of replicates available ($\le 6$) could lead to an incorrect estimation of the uncertainty, and therefore to a poorly estimated bound.

In Suppl. Fig. S1 we visually contextualized all the correlations in function of the theoretical bound, showing that for certain elements (i.e. Co59, Zn66,Fe57 in seed and Mo98, Zn66, Na23, Cu65, Cd114 in leaf), predictions are quite unsatisfactory and this issue is not directly due to data-related issue (i.e. relatively high experimental uncertainty with respect to the overall phenotype variation on the available data), since these predictions currently are $\le 50\%$ of the upper bound. The reason for these low correlations might be due to particularly complex biological mechanisms that regulate these elements.

We also analysed the role of the correlation between the predicted ionome elements concentrations with the prediction performances achieved each element. Suppl. Figs. S56–S58 show that there is a slight positive correlation ($0.38\le r \le 0.54$) between the traits and the prediction performance, indicating some degree of synergistic effect between similar traits during the multi-task learning.

### Prediction performance in function of the model complexity

Table 1 provides a global view of the prediction performance in seed and leaf ionomes in relation to the model complexity, represented by the number of trainable parameters (weights). To obtain a single score, we averaged the intra-country Pearson correlations shown in Fig. 2 across the 18 ionome elements. Overall, the best performing model is Galiana, followed by the fine tuned TL methods. These approaches have nevertheless the highest number of trainable weights (1.4 million). The frozen TL feature extractor methods have the lowest number of parameters, but performances are significantly lower, very close to random chance. Our BMSNN models, on the other hand, drastically reduce the number of parameters with respect to Galiana by using respectively only 6.8% and 11.3% of its number of weights, obtaining performances that are around 14% lower on the seed ionome, and between 7% worse and 10% better on the leaf ionome. Suppl. Table S1 shows the correlation between the model predictions and the heritability of the ionome elements, as reported from^26^.

**Table 1:** Table showing the overall prediction performance in function of the number of weights in the models.

Model	Seed Pearson (std)	Leaf Pearson (std)	# parameters
Galiana	0.202 (0.079)	0.196 (0.090)	1443747
transf50epochs	0.202 (0.085)	0.217 (0.093)	1,443,747
transf30epochs	0.180 (0.070)	0.225 (0.091)	1,443,747
frozen200	0.095 (0.069)	0.100 (0.055)	17,836
frozen500	0.105 (0.065)	0.138 (0.057)	44,536
BMSNN100	0.173 (0.084)	0.216 (0.083)	163,996
BMSNN50	0.172 (0.083)	0.182 (0.097)	98,396
GPSNN	$-0.012$ (0.075)	0.021 (0.051)	21,036

To make sure the hyper parameters we used (see “Methods” “Implementation and validation”) were not sub-optimal for the task, we ran an additional series of nested-cross validations using Optuna^37^ to select the optimal hyper parameters for each model (see Suppl. Mat. Section S5 for more details). The results are shown in Suppl. Table S2 and in Suppl. Figs. S52–S54. They indicate that only Galiana would have slightly benefit from such optimization. These generally underwhelming results can be explained by the fact that the hyper parameter selected by Optuna are probably optimal for the small portion of samples used to optimize them (80% * 20% = 16% of the total, see Suppl. Mat. Section S5), and a degree of overfitting of these hyper parameters happened. Hyper parameter optimization is indeed particularly useful on large datasets, where a reasonable portion of the samples can be spared for that task, while here we are dealing with the opposite problem (dataset underdetermination). Moreover, it is necessary that the validation set used to evaluate the optimization of the hyper parameters is representative of the same population as the test samples. In our case, this condition might not hold due to the small sample size of the validation set coupled with the striking population stratification affecting the AT samples (see “Discussion” for an in-depth analysis of this phenomenon).

### Visualizing the intra-country dynamics of the predictions

So far we have shown aggregate performances to compare the predictions of the different approaches we propose and to investigate the relationship between the model complexity and the prediction performance. In Fig. 3 we focus on the predictions provided by the BMSN100 model, which has the best accuracy in relation to the number of weights (see Table 1). We focus on the 15 best predicted ionome elements within specific countries, ranked by Pearson correlation (top left to bottom right of Fig. 3). As a comparison, Suppl. Fig. S2 shows the 15 best predicted elements by Galiana model, which has 8.8 times more weights.

Even though overall Pearson correlation coefficients shown in Table 1 and in Fig. 2A are very low, corroborating our previous findings^5^ that the concentrations of mineral elements in AT tissues were among the hardest phenotypes to predict, Fig. 3 showed that for certain elements and tissues the model can actually provide relatively good predictions, such as Sr88 in leaf (Italy and UK), Se82 in seed (Italy and Spain) P31 (USA and Sweden), As75 in leaf (USA and UK).

## Discussion

### Population stratification and its impact on genome and ionome distributions of AT

Population stratification is known to be of primary importance in genetic studies^41,42^ and genomic prediction^43^, and it clearly affects also the genotype distribution of AT, since its local adaptation is affected by various geographical cues^44^, including altitude^45^, climate^46,47^, and the biotic environment^48^.

To qualitatively investigate this aspect, in Suppl. Fig. S3 we show the two-dimensional representations of the AT genomes (left plot) and the ionomes (right plot), obtained with t-SNE. Coloring the samples by country clearly shows that certain countries form clusters, with the biggest ones being Sweden (blue) and Spain (red). The country-based clustering is not as striking for what concerns the ionome.

To quantitatively show the extent of the population stratification at the genome level, we trained a multi-class version of Galiana NN model to predict the country of provenience of the AT samples from the genome. We considered the seven major nations shown in Suppl. Fig. S3. F1 and Matthews Correlation Coefficient (MCC) scores are respectively 0.84 and 0.86, indicating that the signal is very strong. Similarly, we built another model to predict the country of origin from the ionome of each AT sample (multi-class prediction, 7 classes), obtaining an MCC of 0.52 and a F1 score of 0.61. Even if in the right panel of Fig. S3 the clustering is less evident, this shows that the correlation between ionome distribution and country is quite strong. The per-country distributions of ionome elements are shown in Suppl. Figs. S16–S50.

#### Population stratification prevents generalization across countries

To investigate the role of population stratification for the prediction of the ionome, in Suppl. Fig. S13 we run an additional evaluation of the performance. While in Fig. 2 the cross-validation (CV) folds were randomly selected (random CV), here we stratified them by country, thereby ensuring that samples from a nation ${\mathcal {N}}$ could be present either in the training or in the test, but never in both (per-country stratified CV). Suppl. Fig. S13 shows the comparison between the prediction scores obtained with the random CV previously shown in Fig. 2 (red and magenta lines), with variations of the Galiana model trained with the per-country stratified CV. We can see that the performance obtained in country CV are significantly lower, indicating that it is difficult to generalize between countries when data from one specific country are not included in the training set. This is related to the difficulty of making predictions beyond the training space^49^ and it is likely due to the fact that AT is characterized by a stronger population structure at the local level with respect to the continental level^44^. This lack of generalization is visible on all the predictions of all elements, but it is more striking on K39, Ca43, Li7, S34 on seed and Mo98, Sr88, K39, Rb85, Ca43 and Na23 on leaf. This validation based on the per-country stratified CV can also be considered the most realistic estimate of the performances that Galiana-like GI models could achieve in real-life scenarios in which they are tasked to predict the ionome of AT samples coming from previously unseen populations.

To test whether this lack of generalization is due to some bias related to the distribution of variants in different countries, in Suppl. Fig. S13 we tested different data preprocessing methods. The blue line represents the input data standardization we used so far (see “Transforming AT WGS data into NN-understandable input tensors”). The green line indicates that no standardization is performed (the NN input are the raw integer counts of the 17 types of variants mapped on each gene). The yellow line indicates that instead of standardizing the variant occurrences across the entire dataset, we normalized the counts within each gene independently, ensuring that the frequencies of the 17 types of variants mapped on each gene sum to 1, thereby removing the effect of genes that might be preferentially mutated in different countries. Suppl. Fig. S13 and Suppl. Fig. S4 show that there is no striking difference between these preprocessing approaches, and that the major cause for the performance discrepancy is indeed the CV strategy.

To investigate this aspect further, we filtered out low-frequency and rare variants, to focus on common variants as candidate alleles undergoing positive selection^50^. In Suppl. Fig. S5, we filtered out variants with Minor Allele Frequency (MAF) $\le 5\%$ and $\le 10\%$ from our input tensors. Similarly to Suppl. Fig. S13, Suppl. Fig. S5 indicates that in this case MAF does not seem to affect the generalization between countries.

#### Population stratification affects the model validation

Supplementary Fig. S14 shows the performance of our GI models when their performances (correlations) are computed over the entire dataset instead of computing the average intra-country correlation like in Fig. 2. We can see that the correlation achieved for most elements is higher, and that now the results of the GPSNN model (which does not use any genetic information) are much closer to the genetics-based methods. This is due to the fact that, due to the country-specific ionome concentrations (see Suppl. Fig. S16–S50), if the models are evaluated on the entire dataset, even predicting a country-specific *average* ionome can lead to an apparently relatively high model performance. What we see here is the genetic version of the Simpson’s paradox^51^: the accuracy (correlation) in the entire dataset is deceptively good, even though the intra-country correlations are moderate-to-poor. An explanation to this behavior is that since we now evaluate on the overall Pearson on the entire dataset, instead of averaging the predictions obtained within the seven major countries, most (or at least a greater proportion) of the variance that needs to be explained by the model is now *geographic* and no longer genetic. But because this is a trivial task (most models manage to do that, including the GPSNN), the models almost maximize performance. And when performance is close to the maximum (in terms of the theoretical upper bound), we have 50% chance of exceeding it, because we can see the Pearson prediction score as a random variable centered on the upper bound.

When analysing heterogeneous data from multiple populations and/or geographic locations, stratification must be carefully accounted for. In the modeling phase, ignoring stratification can indeed lead to model misspecifications and spurious results, particularly when looking for causal variants^52^. When designing the evaluation strategy, it is important to choose the procedure according to the *true* prediction objective (e.g. prediction of future observations, predictions across-environments etc.), otherwise there is a risk of over-estimation of the model performance^51,53^.

To confirm that the Simpson’s paradox is causing the results shown in Suppl. Fig. S14, we ran additional analysis. In Suppl. Fig. S6 we show that, when the population stratification effect is removed by computing intra-country performances, the GPSNN is consistently overmatched by the genetic-based models ($p = 1.6\times 10^{-21}$ on seed and $p = 5.2\times 10^{-17}$ on leaf). To give a visual intuition of the mechanism that allows GPSNN or similar country-based predictors to achieve *apparent* competitive performances when evaluated on the entire dataset, in Suppl. Fig. S7–S10 we show the scatter plots of the correlations obtained on the entire dataset. We can see that while Galiana and BMSNN100 points still have an elongated shape, GPSNN predicts vertical series of dots (the same concentration for all geographically close AT samples). The global correlation arises from the fact that the barycenter of these lines happens to be on a population-dependent slope (see Fig. S7), as described by the Simpson’s paradox. This behavior is even more pronounced when looking at the intra-country scatter plots (see Suppl. Fig. S11), compared to those obtained by genetics-based models (see Fig. 3 and Suppl. Fig. S12).

## Conclusion

In this paper we proposed novel NN GI models to address the widespread underdetermination of genomic datasets, and we applied them to the prediction of leaf and seed ionome elements concentrations in AT. We show that the sparsity of biological networks can be used to build sparse models with reduced number of parameters that can still compete with larger dense NN models. We also investigate the effects of population stratification in AT, showing how it influences the evaluation of the performance and the model generalization ability.

### Supplementary Information

Supplementary Information 1.

Supplementary Information 2.

## Data Availability

*A. thaliana* leaf and seeds were not involved in the study. We used the third-party data described in the “Availability of data and material” item below. The code is available at https://bitbucket.org/eddiewrc/ionomeprediction. The datasets analysed during the current study are available at the following URLs. The WGS AT original data is available at https://1001genomes.org/. The ionome data is available at https://ffionexplorer.nottingham.ac.uk/ionmap/.

## Code availability

The code is available at https://bitbucket.org/eddiewrc/ionomeprediction.
