# Supplementary material for: Biologically meaningful genome interpretation models to address data underdetermination for the leaf and seed ionome prediction in Arabidopsis thaliana
Source: Sci Rep. 2024 Jun 8;14:13188. doi: 10.1038/s41598-024-63855-6 (PMC11162433; doi:10.1038/s41598-024-63855-6)
Supplement: Supplementary file 2 — Supplementary Information 2. [file 41598_2024_63855_MOESM2_ESM.pdf]

Supplementary Material

April 10, 2024

S1 Supplementary Tables

Model	Seed	Leaf
galiana	0.84	0.65
transf50	0.76	0.77
transf30	0.83	0.71
frozen200	0.53	0.29
frozen500	0.50	0.38
BMSNN100	0.78	0.66
BMSNN50	0.75	0.75
GPSNN	0.61	-0.05

Table S1: Table showing the correlations between the intra-country performance of each model on the ionome elements heritability reported in Campos, et al. (2021).

S2 Supplementary Figures

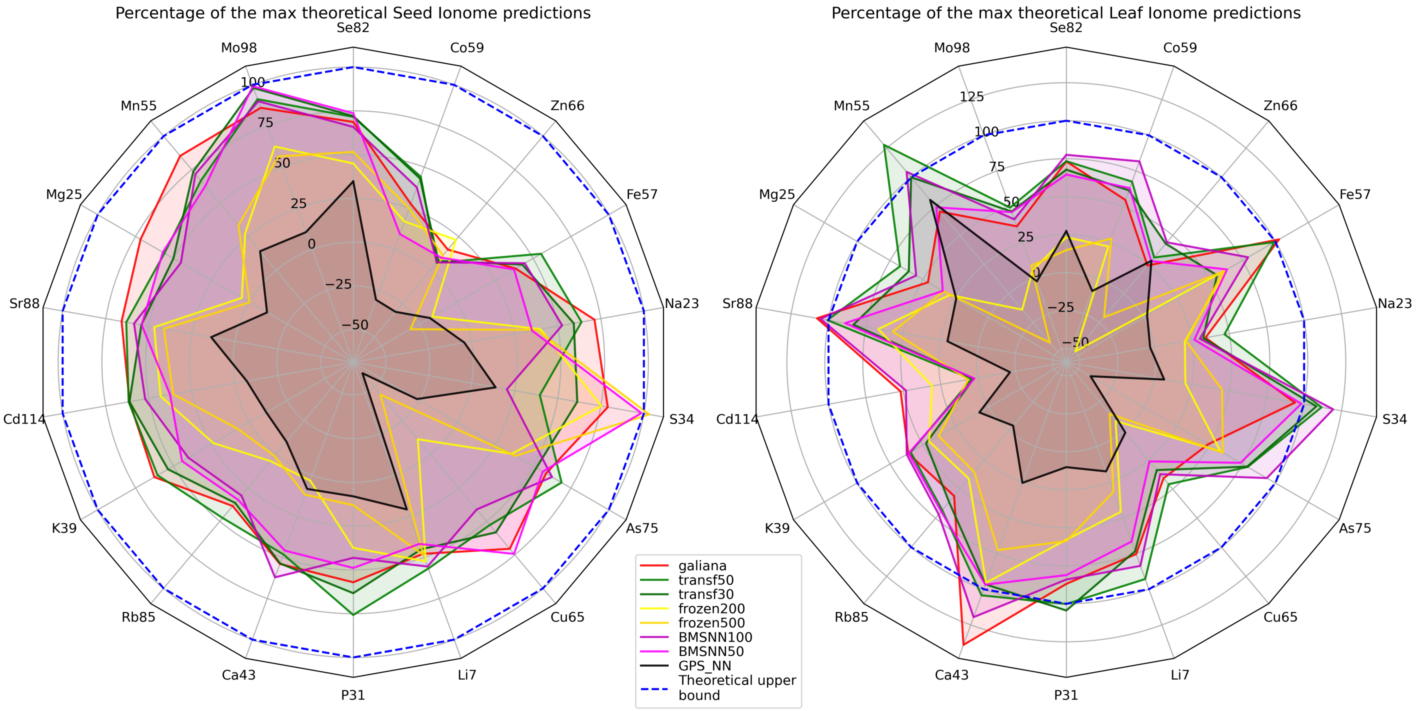

Figure S1: Radar plot showing the performance of the models tested presented as percentage of the theoretical Pearson correlation upper bound.

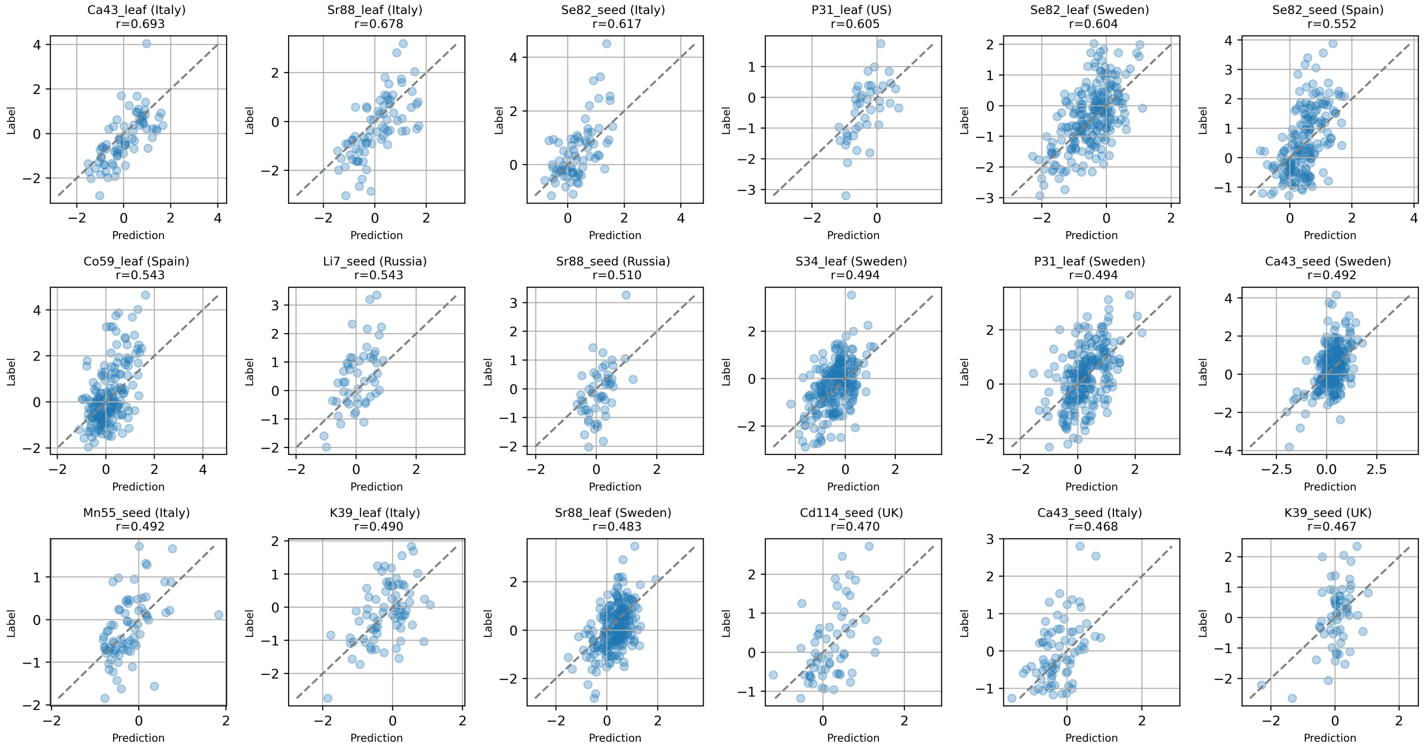

Figure S2: Scatter plots of the best 15 intra-country predictions obtained with the Galiana model.

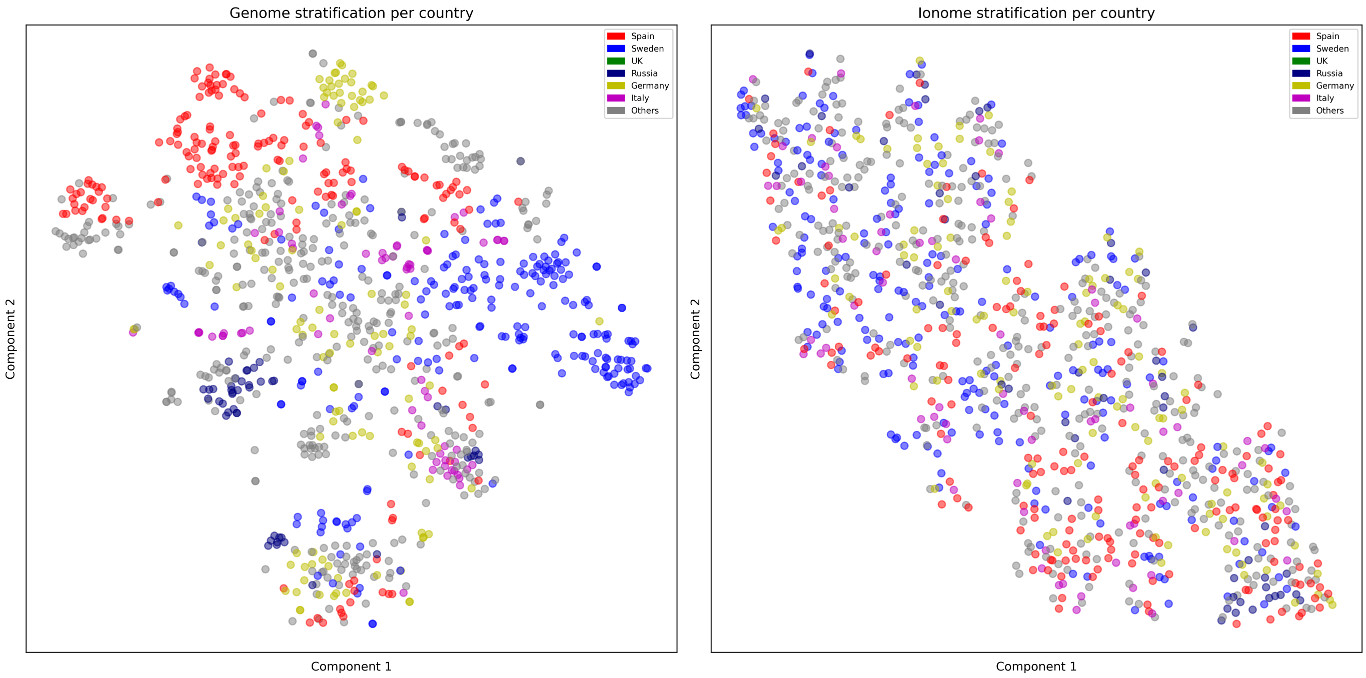

Figure S3: Scatter plots the 2-dimensional t-SNE visualization of the distribution of AT genomes (left) and ionomes (right), colored by country.

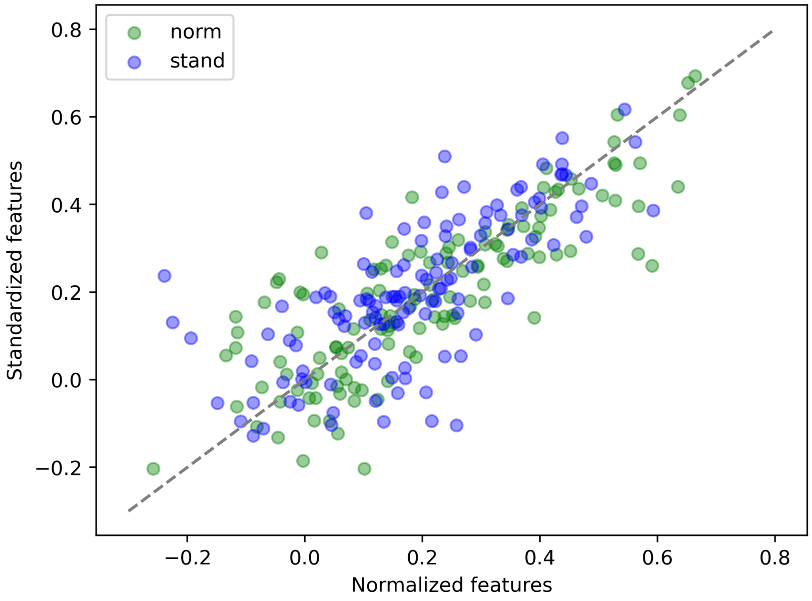

Figure S4: Scatter plots showing the performances obtained while standardizing or normalizing the input tensors. Both approaches lead to similar results.

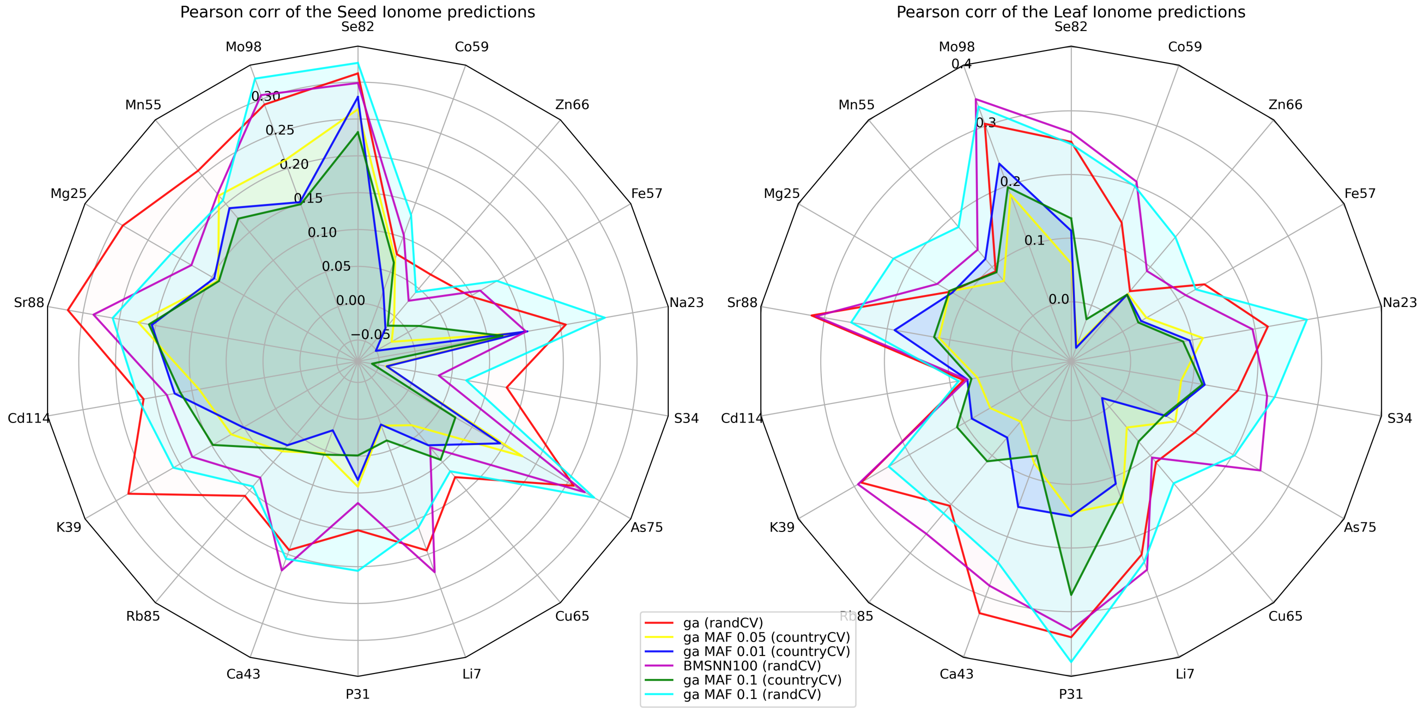

Figure S5: Radar plot showing the performances obtained by filtering the VCF input data with different MAFs (0.01, 0.05, 0.1). The performances obtained using the CV stratified by country (countryCV) are lower, regardless of the MAF. MAF filtering applied on the randCV (cyan line) leads to performance similar to the standard galiana approach. Except for the BMSNN100 model, all the other approaches used Galiana architecture (shortened in "ga" in the plot).

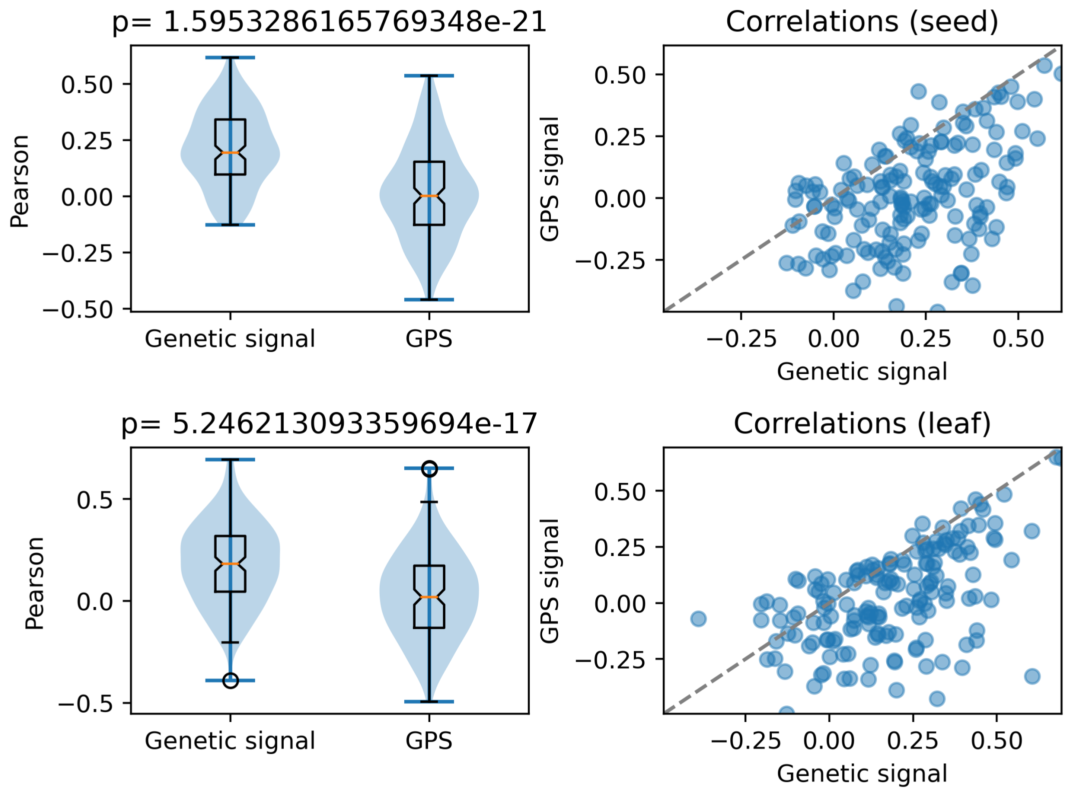

Figure S6: Plots showing that genetics-based models significantly overmatch GPS-based models when predictions are evaluated intra-country.

S3 Best predicted ionome elements over the entire dataset

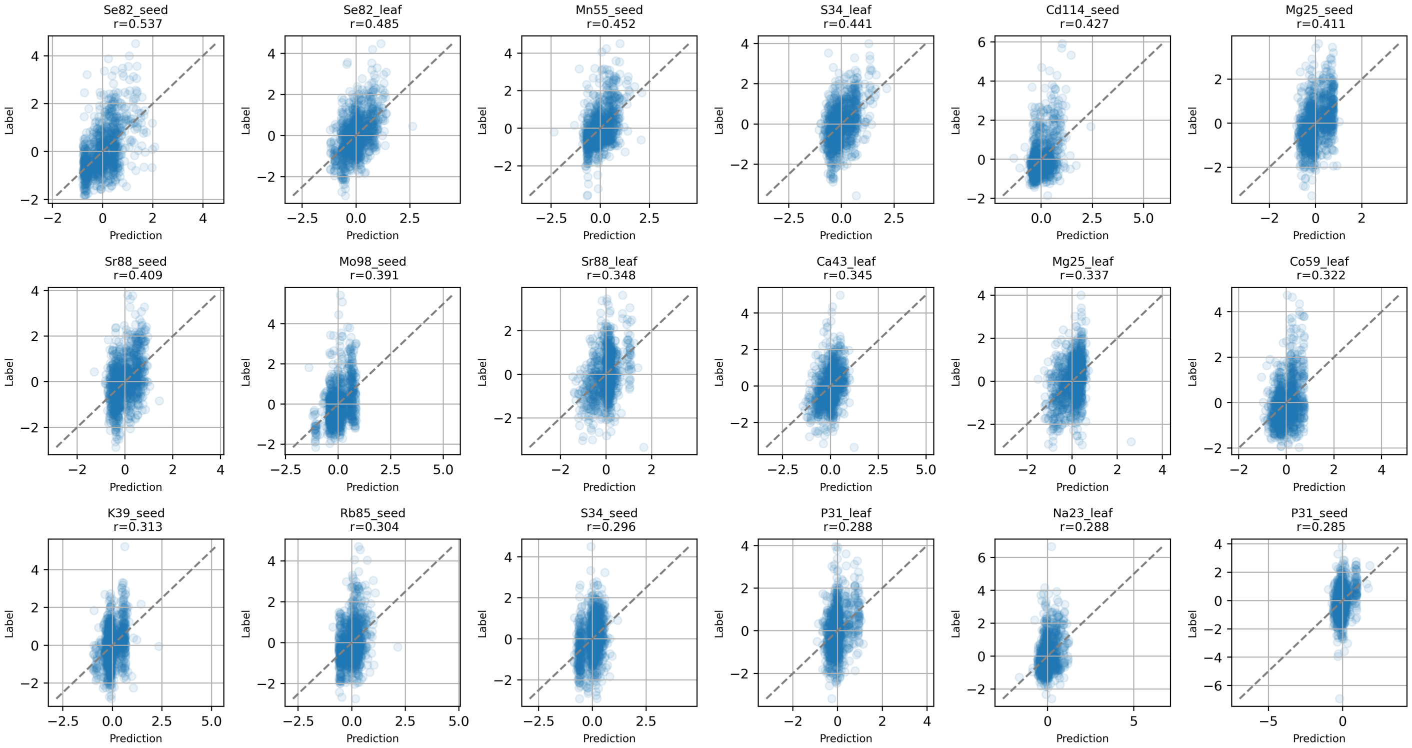

Figure S7: Scatter plots of the best predicted 15 elements over the entire dataset using the GPSNN model trained with a random CV.

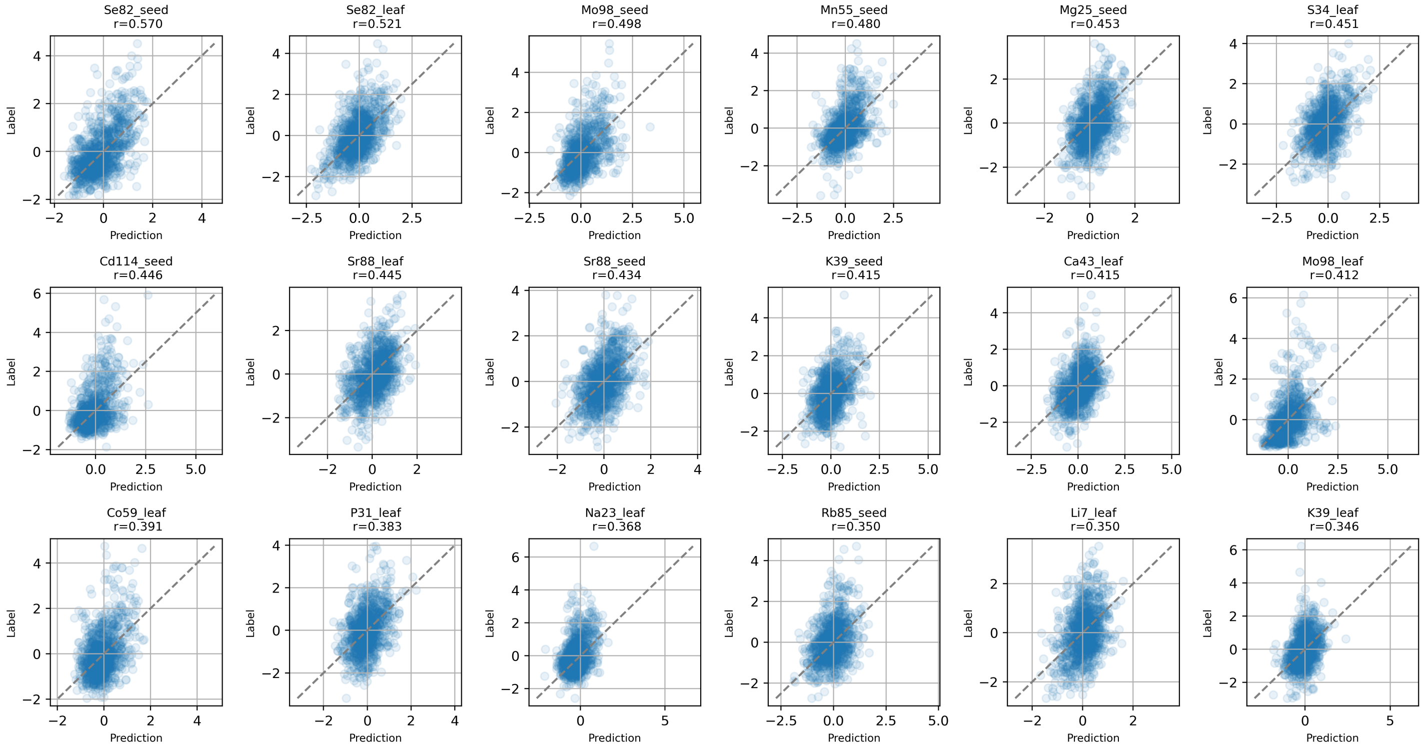

Figure S8: Scatter plots of the best predicted 15 elements over the entire dataset using the Galiana model trained with a random CV.

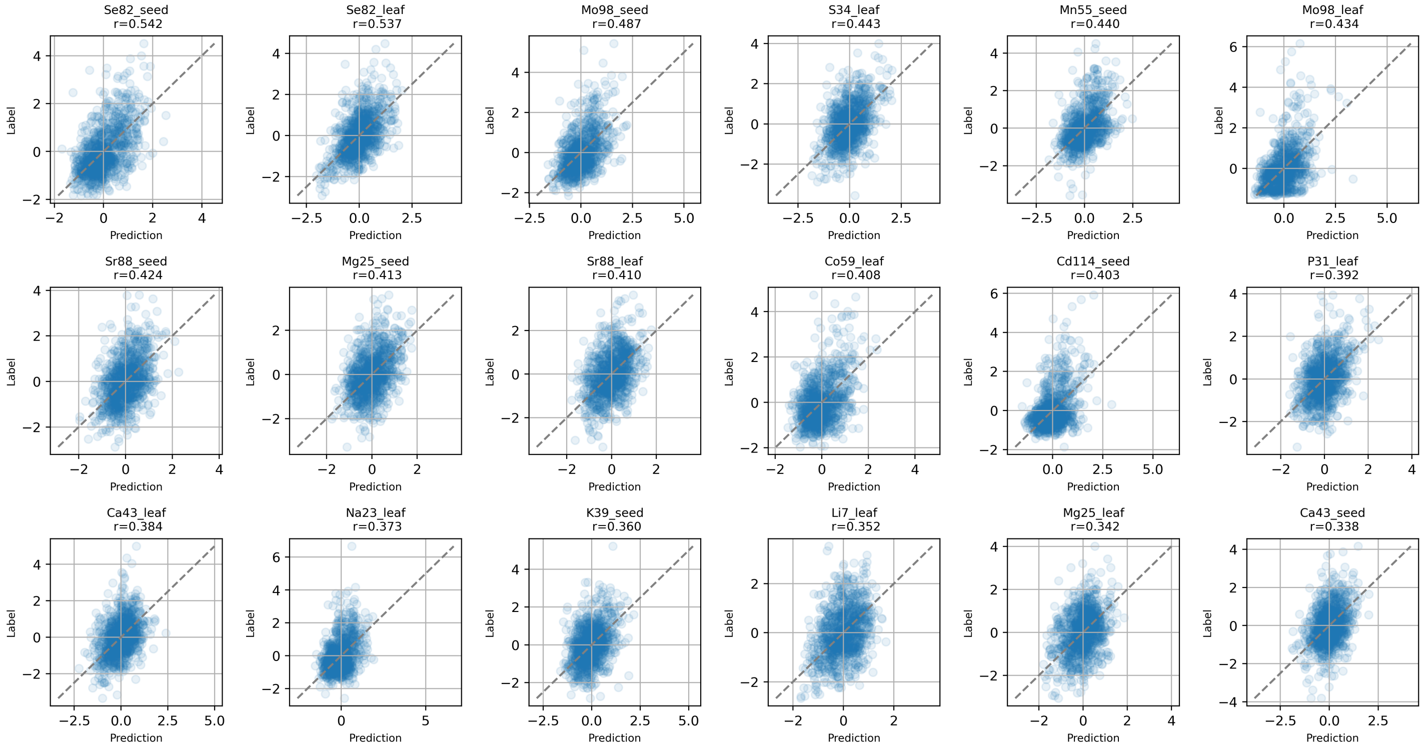

Figure S9: Scatter plots of the best predicted 15 elements over the entire dataset using the BMSNN100 model trained in random CV.

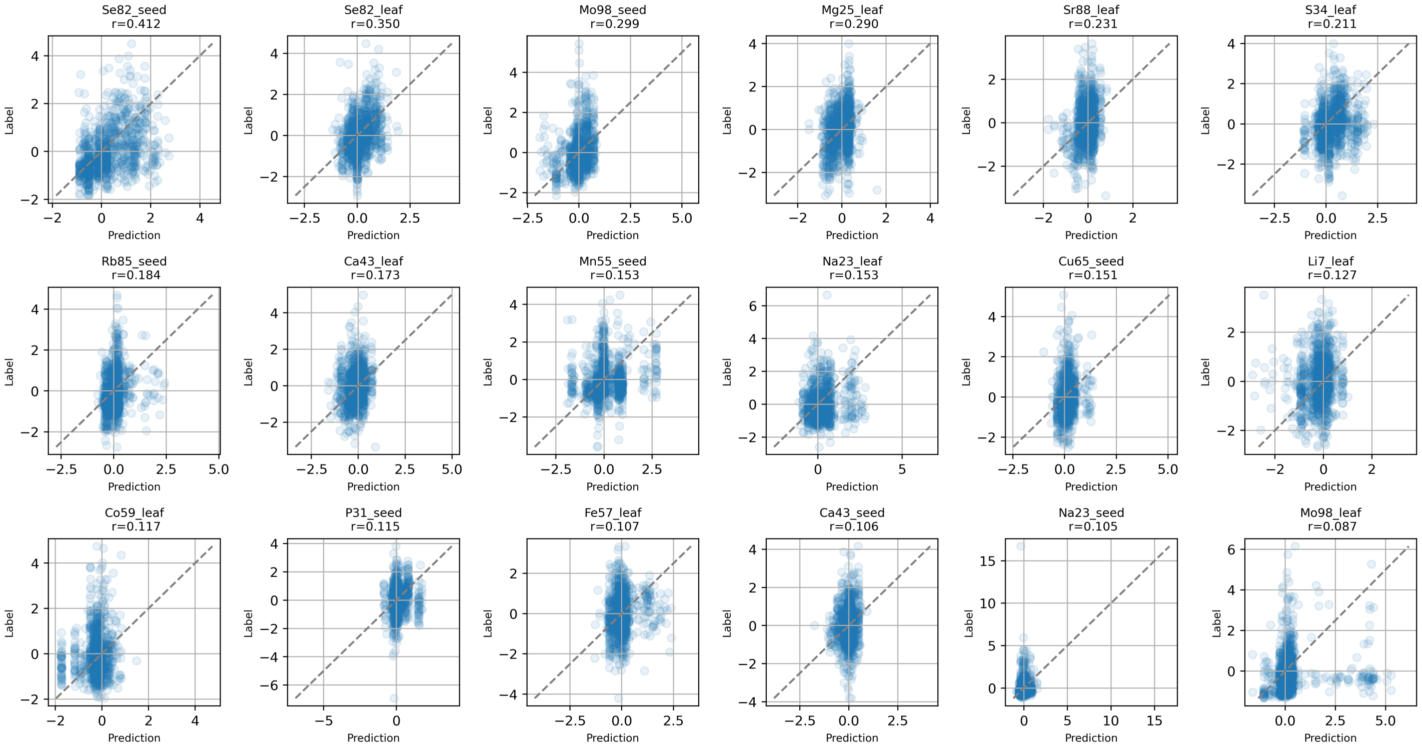

Figure S10: Scatter plots of the best predicted 15 elements over the entire dataset using the GPSNN model trained with cross-validation stratified per country.

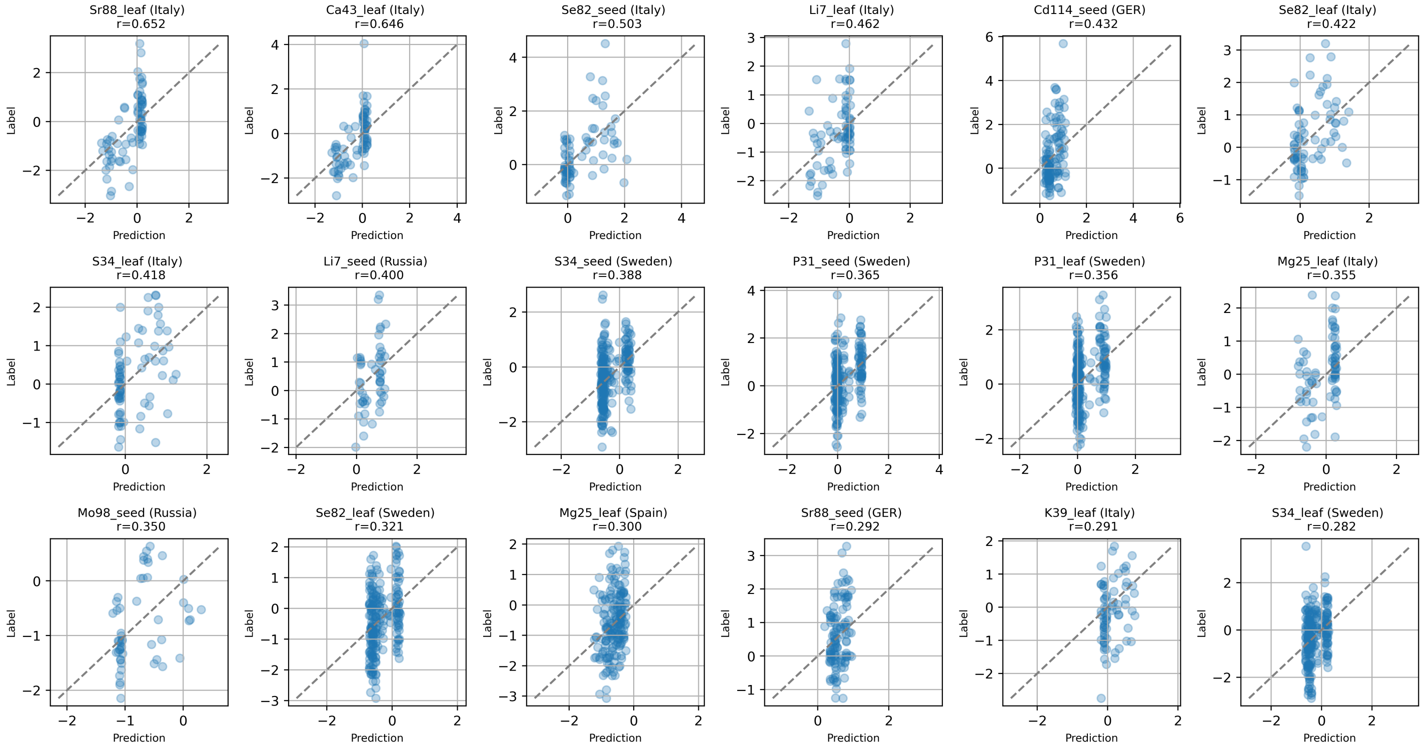

Figure S11: Scatter plots of the best 15 intra-country predictions obtained with the GPSNN model trained with random CV.

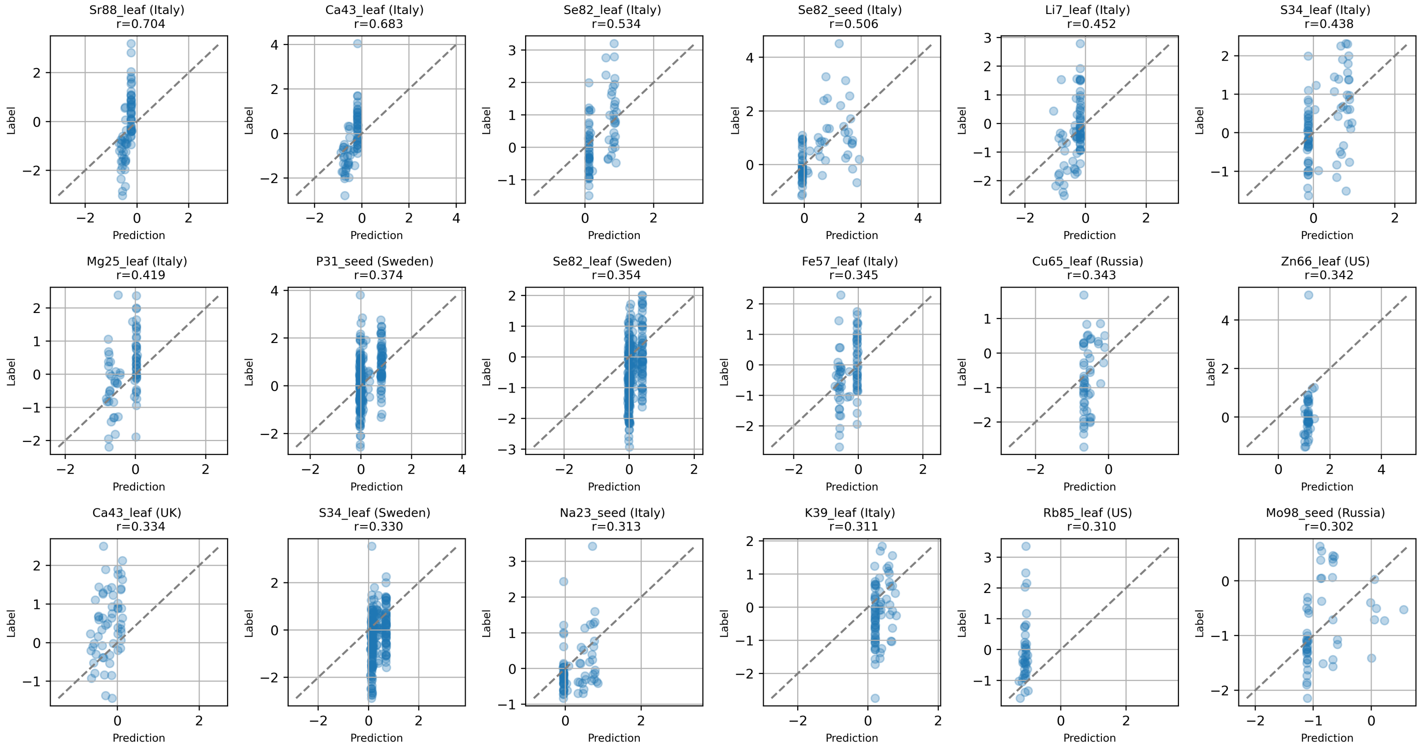

Figure S12: Scatter plots of the best 15 intra-country predictions obtained with the GPSNN model trained with country-stratified CV.

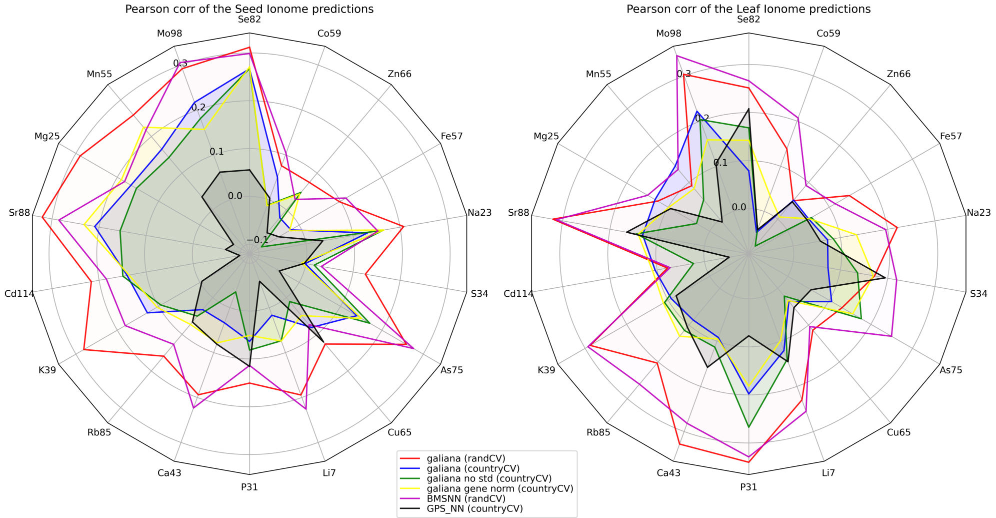

Figure S13: Radar plot showing the dependence of the performance in relation to the type of CV used (random CV or CV stratified per country). Galiana and BMSNN evaluated in random CV are compared with Galiana evaluated per-country stratified CV.

S4 Per-country ionome elements concentrations

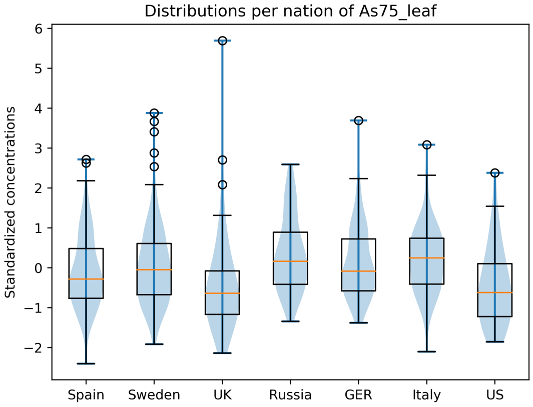

Figure S15: Plot showing the ionome elements concentrations in the 7 major countries.

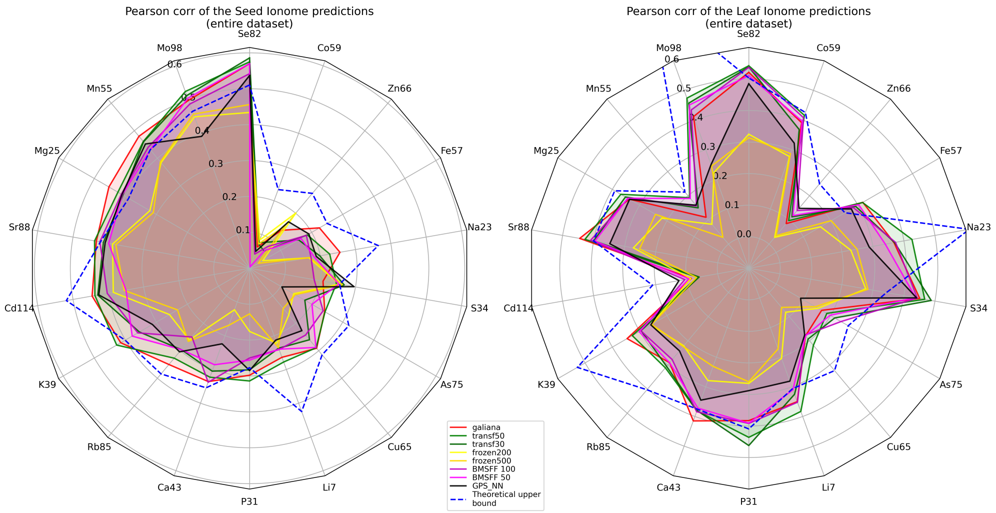

Figure S14: Radar plot showing the random cross-validation (randCV) performance of the models tested presented when they are averaged over the entire dataset instead that being averaged averaged within each country (The intra-country correlations presented in Fig. 2 of the main paper).

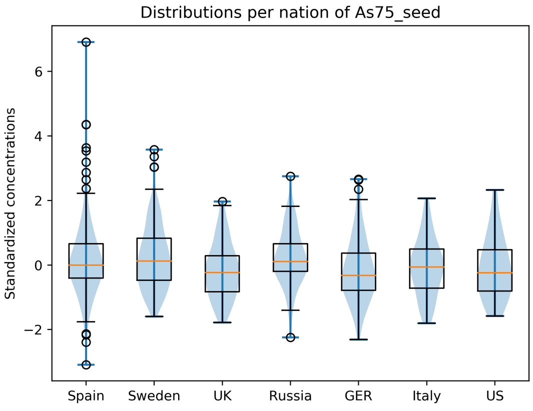

Figure S16: Plot showing the ionome elements concentrations in the 7 major countries.

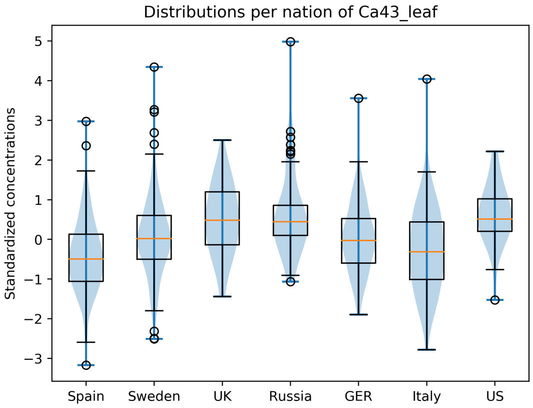

Figure S17: Plot showing the ionome elements concentrations in the 7 major countries.

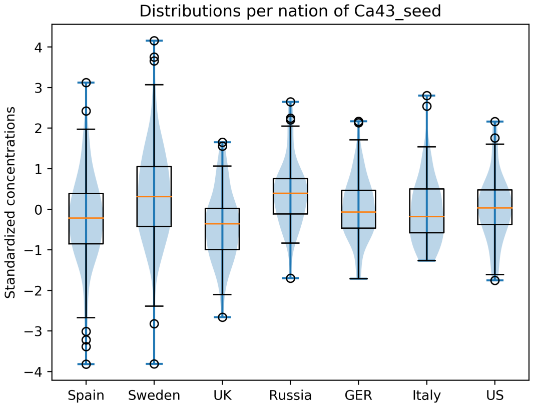

Figure S18: Plot showing the ionome elements concentrations in the 7 major countries.

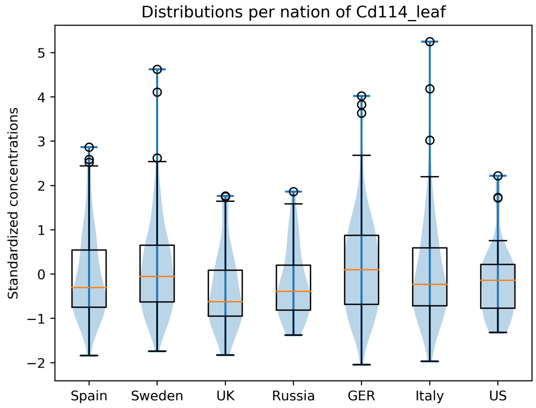

Figure S19: Plot showing the ionome elements concentrations in the 7 major countries.

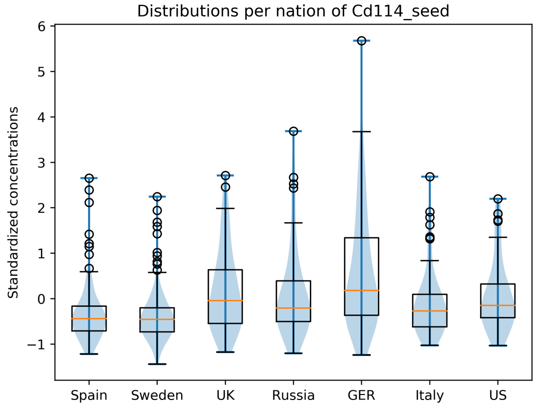

Figure S20: Plot showing the ionome elements concentrations in the 7 major countries.

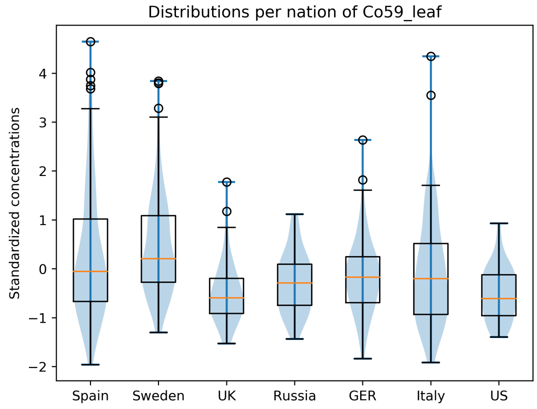

Figure S21: Plot showing the ionome elements concentrations in the 7 major countries.

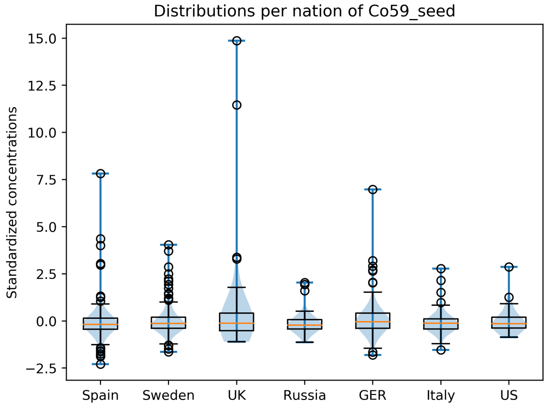

Figure S22: Plot showing the ionome elements concentrations in the 7 major countries.

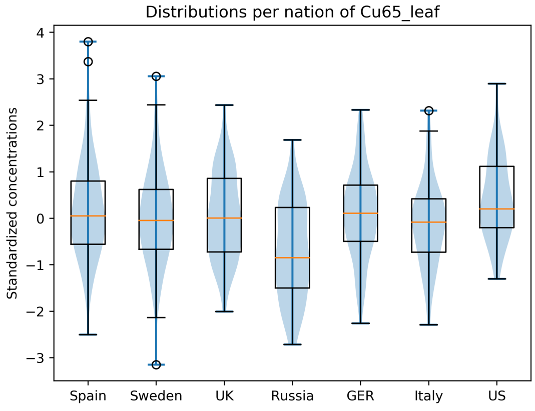

Figure S23: Plot showing the ionome elements concentrations in the 7 major countries.

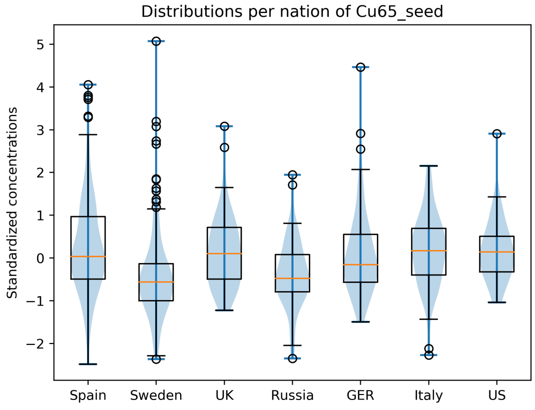

Figure S24: Plot showing the ionome elements concentrations in the 7 major countries.

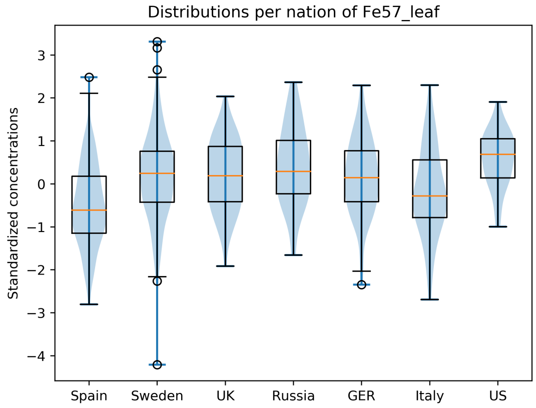

Figure S25: Plot showing the ionome elements concentrations in the 7 major countries.

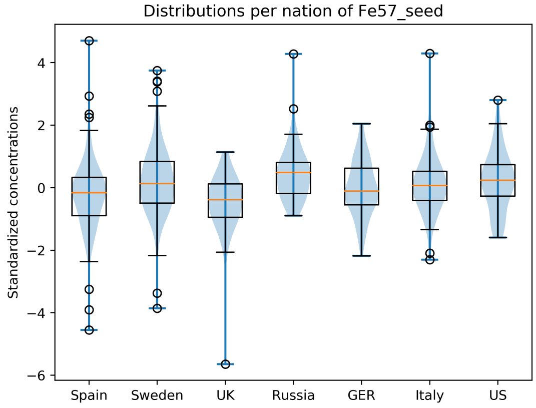

Figure S26: Plot showing the ionome elements concentrations in the 7 major countries.

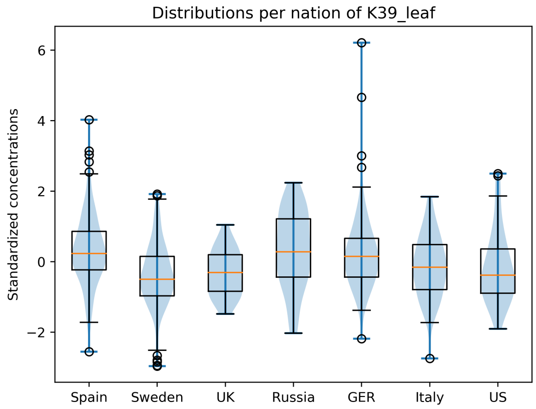

Figure S27: Plot showing the ionome elements concentrations in the 7 major countries.

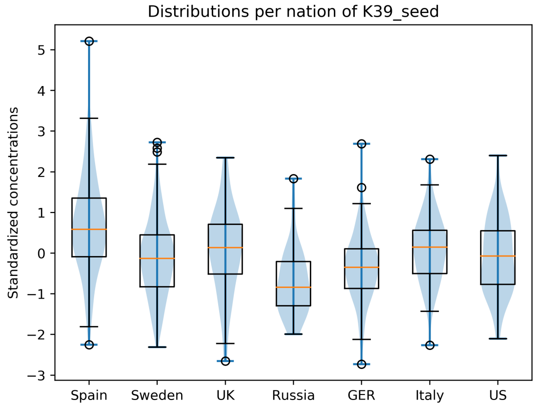

Figure S28: Plot showing the ionome elements concentrations in the 7 major countries.

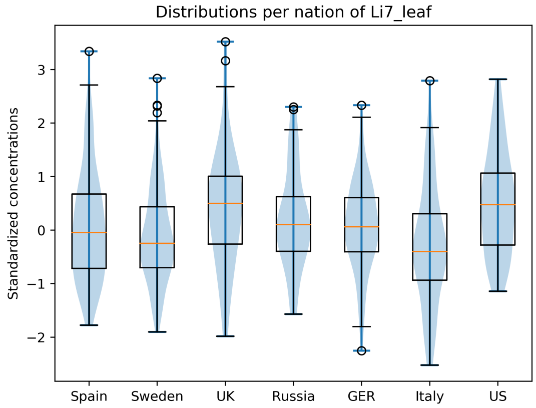

Figure S29: Plot showing the ionome elements concentrations in the 7 major countries.

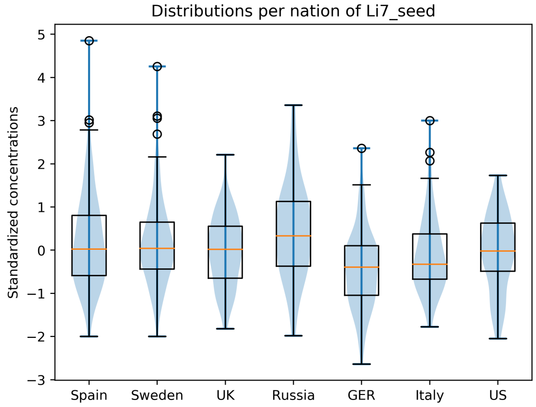

Figure S30: Plot showing the ionome elements concentrations in the 7 major countries.

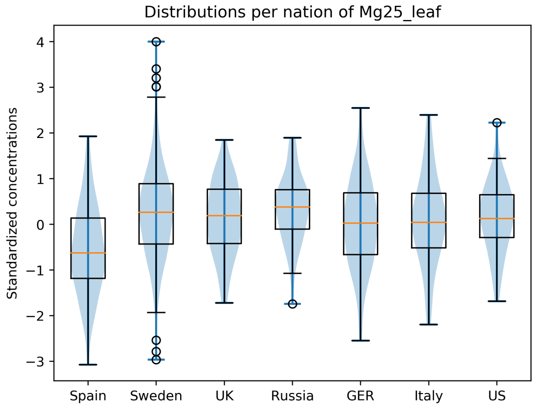

Figure S31: Plot showing the ionome elements concentrations in the 7 major countries.

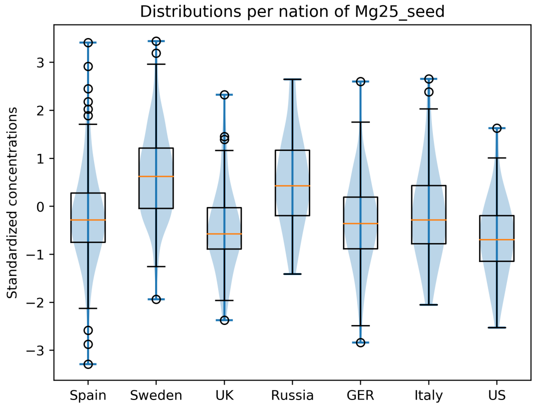

Figure S32: Plot showing the ionome elements concentrations in the 7 major countries.

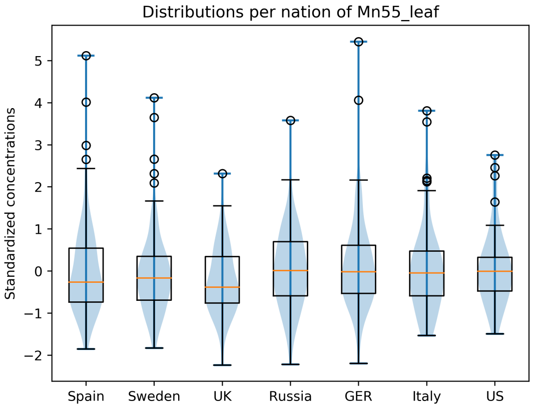

Figure S33: Plot showing the ionome elements concentrations in the 7 major countries.

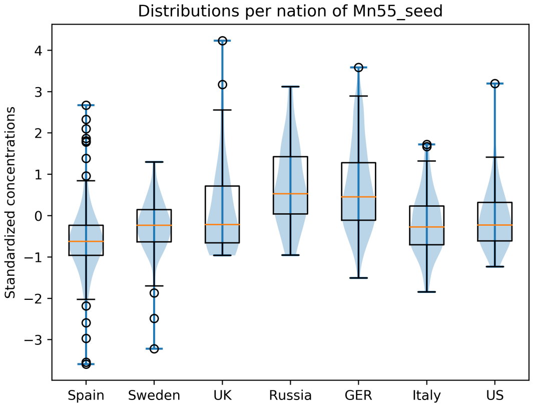

Figure S34: Plot showing the ionome elements concentrations in the 7 major countries.

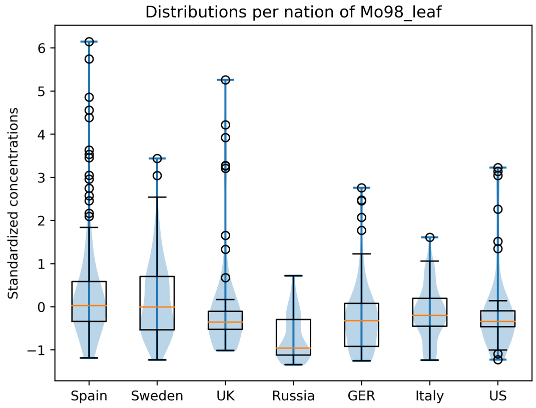

Figure S35: Plot showing the ionome elements concentrations in the 7 major countries.

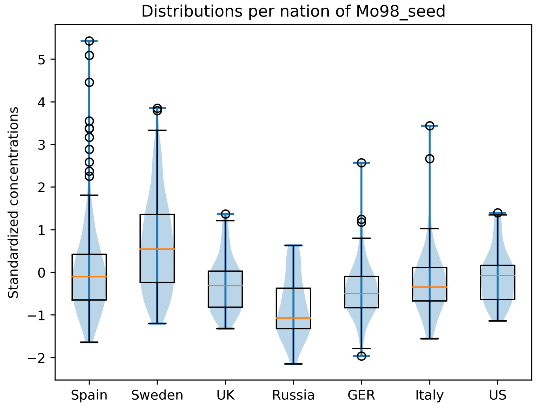

Figure S36: Plot showing the ionome elements concentrations in the 7 major countries.

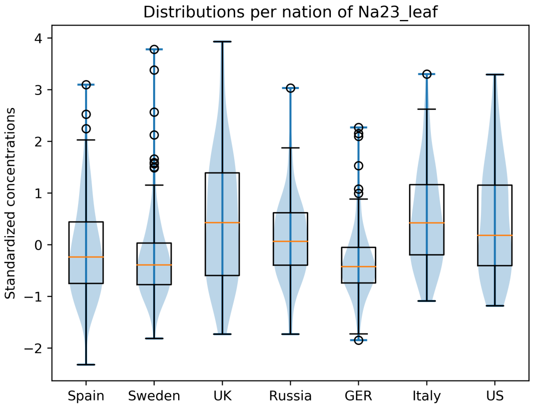

Figure S37: Plot showing the ionome elements concentrations in the 7 major countries.

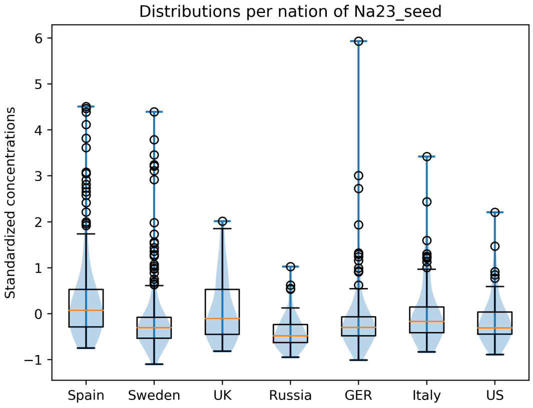

Figure S38: Plot showing the ionome elements concentrations in the 7 major countries.

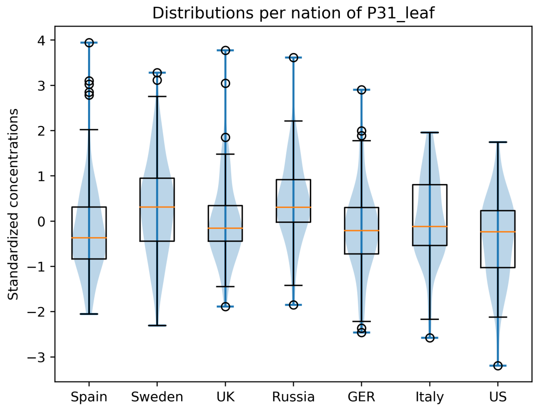

Figure S39: Plot showing the ionome elements concentrations in the 7 major countries.

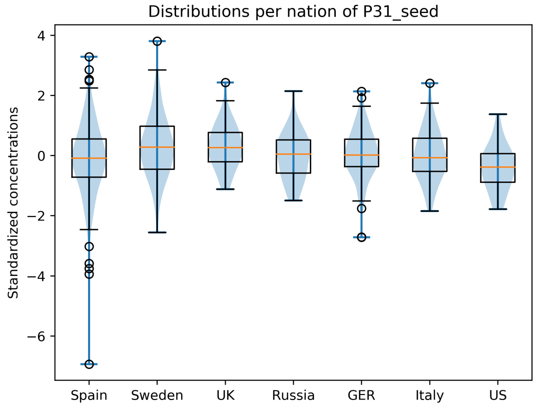

Figure S40: Plot showing the ionome elements concentrations in the 7 major countries.

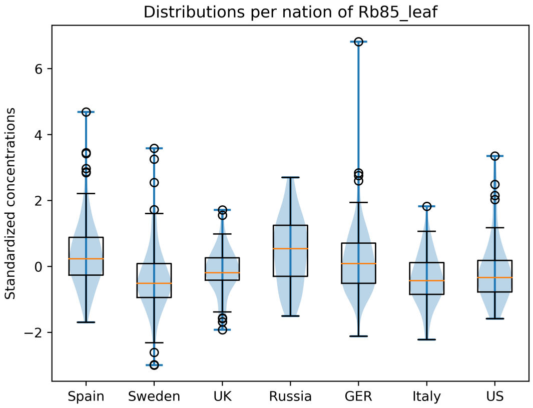

Figure S41: Plot showing the ionome elements concentrations in the 7 major countries.

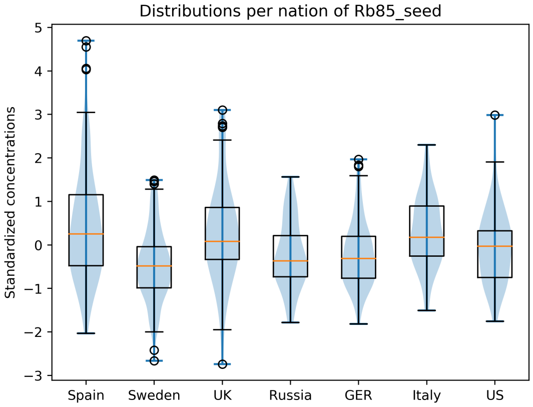

Figure S42: Plot showing the ionome elements concentrations in the 7 major countries.

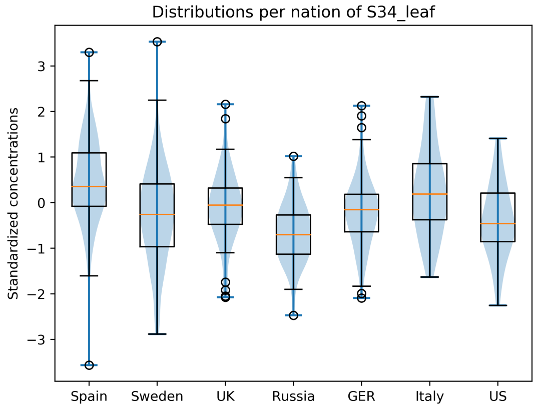

Figure S43: Plot showing the ionome elements concentrations in the 7 major countries.

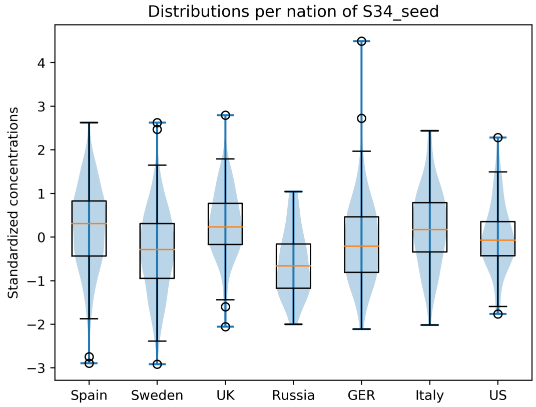

Figure S44: Plot showing the ionome elements concentrations in the 7 major countries.

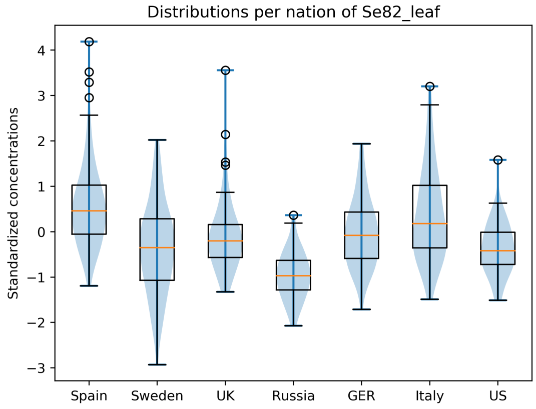

Figure S45: Plot showing the ionome elements concentrations in the 7 major countries.

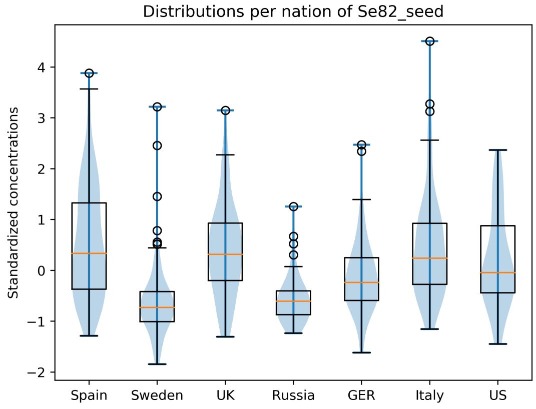

Figure S46: Plot showing the ionome elements concentrations in the 7 major countries.

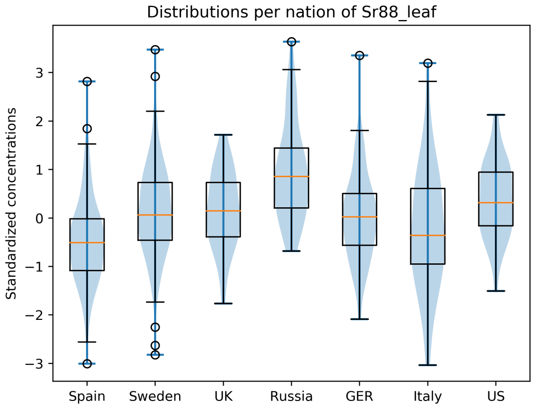

Figure S47: Plot showing the ionome elements concentrations in the 7 major countries.

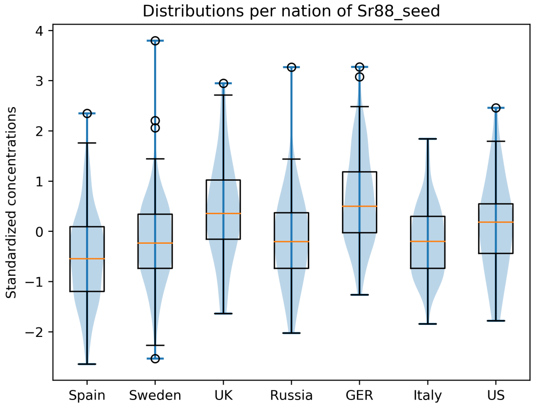

Figure S48: Plot showing the ionome elements concentrations in the 7 major countries.

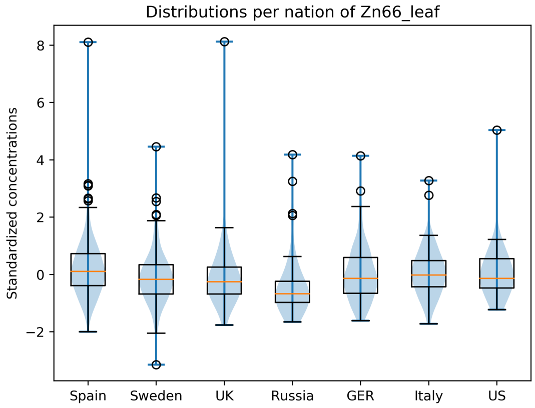

Figure S49: Plot showing the ionome elements concentrations in the 7 major countries.

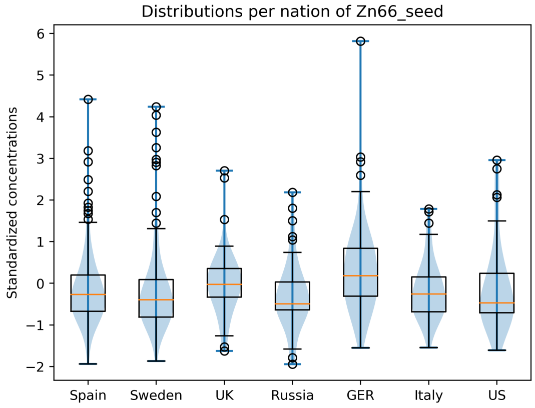

Figure S50: Plot showing the ionome elements concentrations in the 7 major countries.

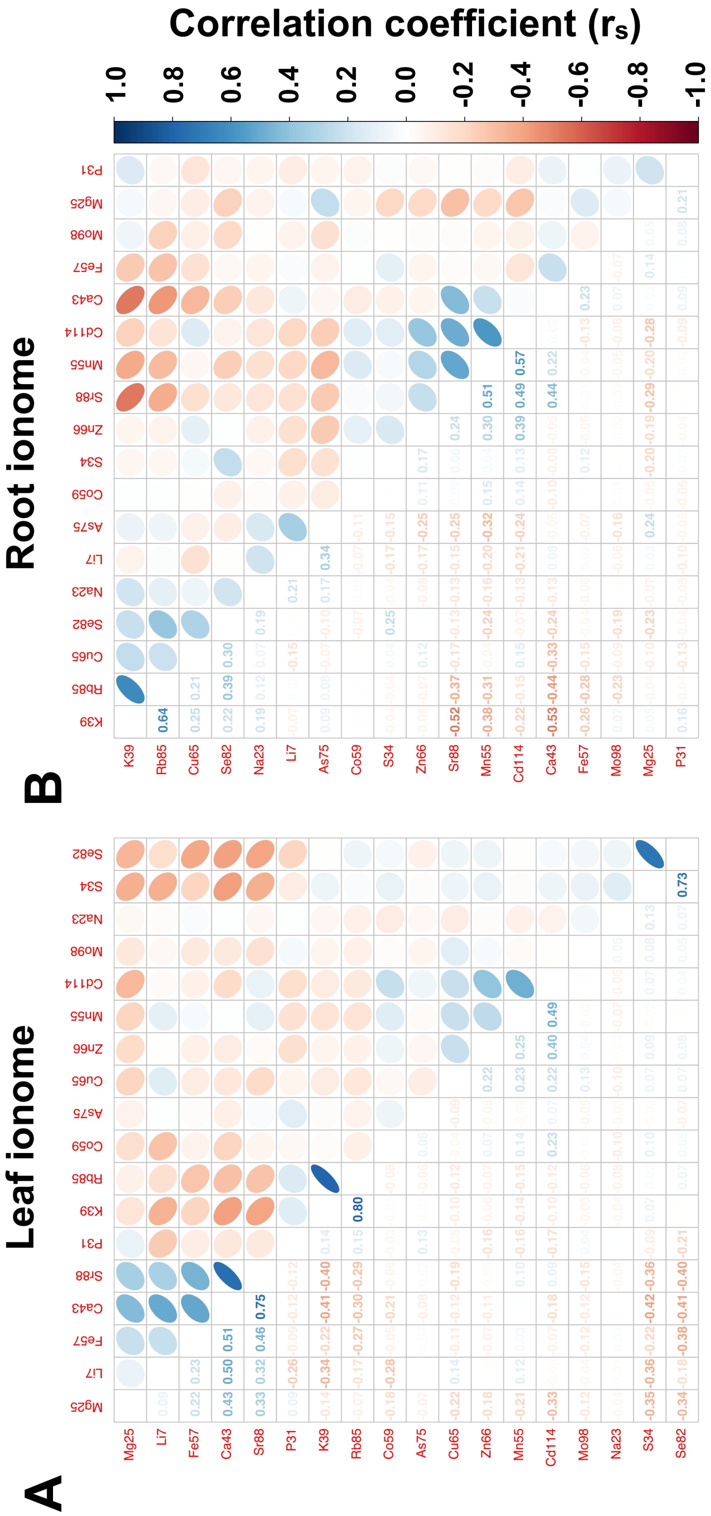

Figure S51: Figure showing the autocorrelations among the ionome elements.

S5 Hyper Parameter optimization

We performed additional nested cross-validations to determine the effect of an Hyper Parameter Optimization (HPO) on the results shown in Table 1 of the main manuscript.

Here we describe the procedure followed to perform this optimization. We used a 5-folds cross-validation. In each of the 5 CV folds, 80% of the samples are used as training set and 20% are used as test set, to evaluate the performance. In each fold, we further divided the training set samples into two parts. The first contains 80% of the training data, and it is used to train the model weights with the Adam optimizer. The second part contains 20% of the training samples (80% $\times$ 20% = 16% of the total), and it is used as a validation set for the hyper parameter optimization. We performed this optimization with the Optuna library and the TPESampler, which performs a Bayesian exploration of the parameter space. In each CV fold, Optuna thus uses 20% of the training set to select the optimal hyper parameters. Once these parameters have been found, we use them to train the model on the entire training set, and we predict the samples in the test set (that have not been used until now in the CV). We then collect the predictions and we repeat this procedure on the next fold. The following table shows the comparison between 1) the predictions performance obtained without hyper-parameter optimization, shown in Table 1 of the main paper, and the performance obtained with the nested hyper parameter optimization. The models showing the performances obtained with the Hyper Parameter Optimization described above, are indicated with the HPO acronym. From this table we can see that Galiana is the only model to have a slight benefit from the HPO. In the BMSNN50_HPO model, the results are very similar in the Seed tissue, and they are lower than the BMSNN50 model without HPO in the Leaf tissue. For what concerns the BMSN100_HPO model, in both tissues the performance is lower.

Model	Seed Pearson (std)	Leaf Pearson (std)	# parameters
Galiana	0.202	0.196	
Galiana_HPO	0.208	0.217	
BMSNN50	0.172	0.182	
BMSNN50_HPO	0.142	0.184	
BMSNN100	0.173	0.216	
BMSNN100_HPO	0.150	0.211	

Table S2: Table showing the difference between the performance obtained without Hyper-Parameter Optimization (HPO), and by performing it with Optuna. We denoted the model in which we performed a nested-cross-validation with Optuna by appending HPO to their name.

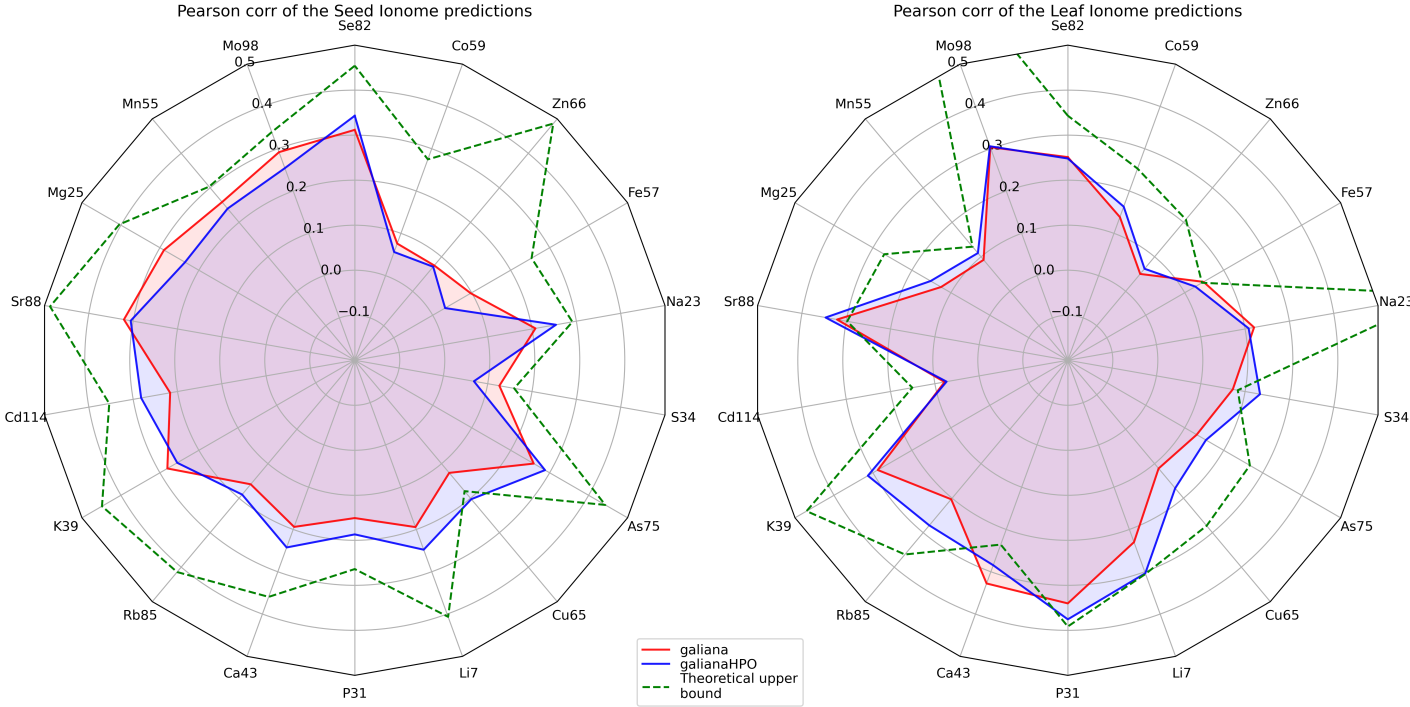

Figure S52: Radar plots showing the comparison betweent he performance obtained by the Galiana model with (red) and without (blue) Hyper Parameter Optimization (HPO).

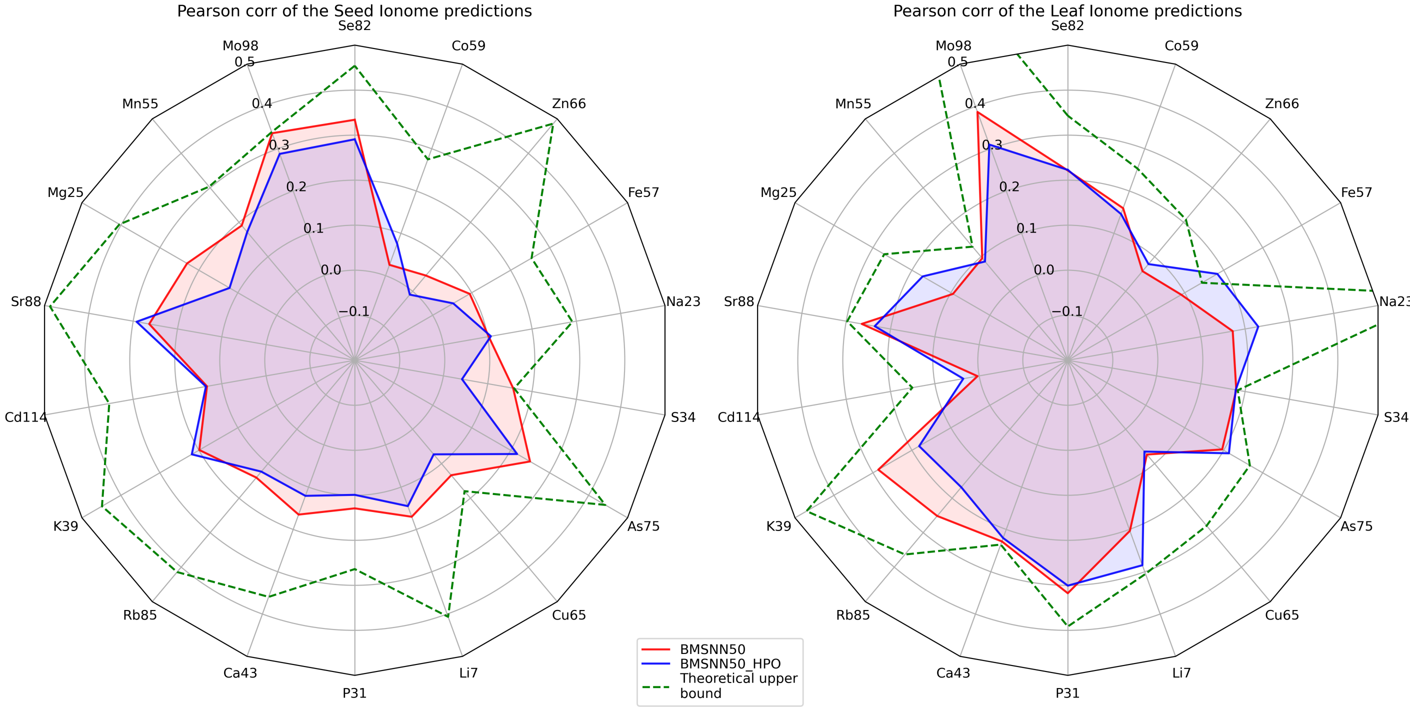

Figure S53: Radar plots showing the comparison betweent he performance obtained by the BMSNN50 model with (red) and without (blue) Hyper Parameter Optimization (HPO).

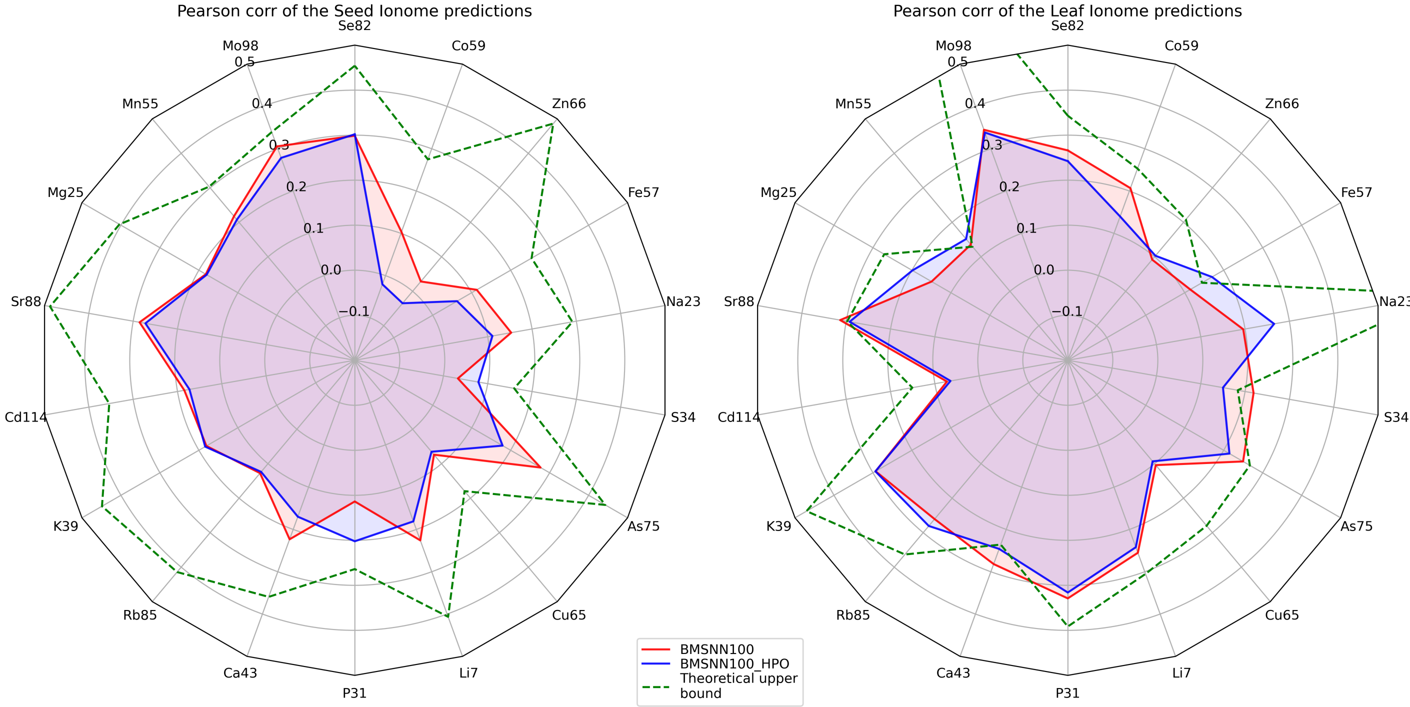

Figure S54: Radar plots showing the comparison betweent he performance obtained by the BMSNN100 model with (red) and without (blue) Hyper Parameter Optimization (HPO).

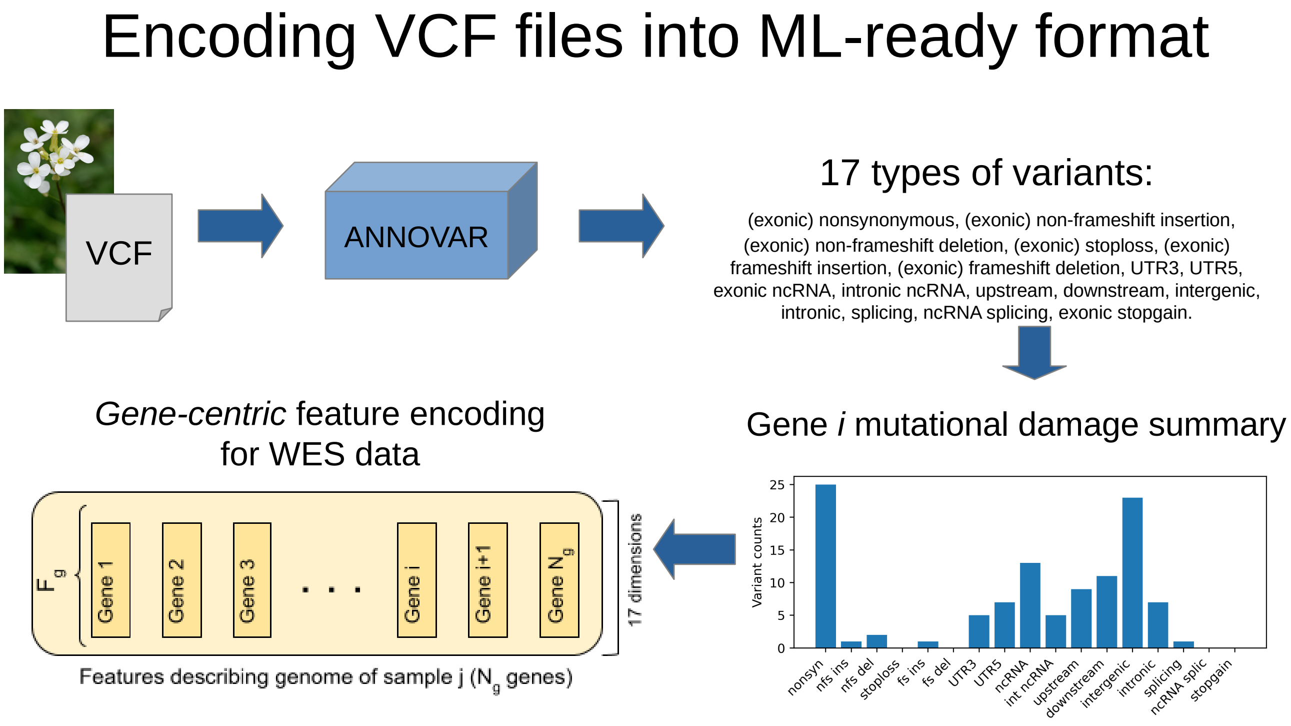

Figure S55: Figure showing the procedure used to trasnfor the AT WGS data into ML-understandable feature vectors.

S6 Analysis of the relation between similarity between traits and prediction performance

We also analysed the role of the correlation between the predicted traits (ionome element concentrations) with the prediction performances achieved on the prediction of each element. The predictions have been computed with the BM-SNN50 model.

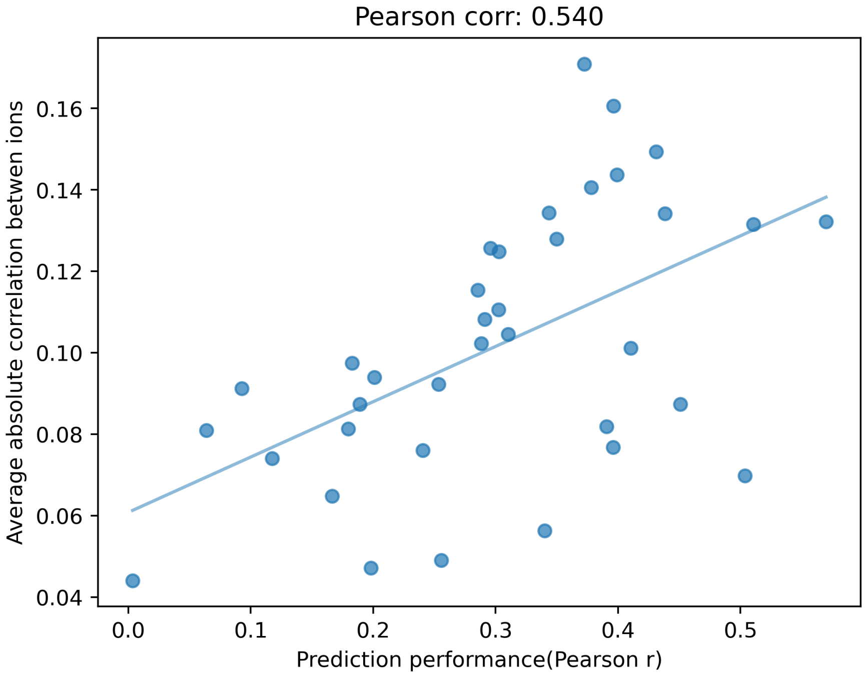

Figure S56: Scatter plot showing the relation between 1) the average absolute correlation between ionome values (y axis) and 2) the average prediction for each ionome element (x axis).

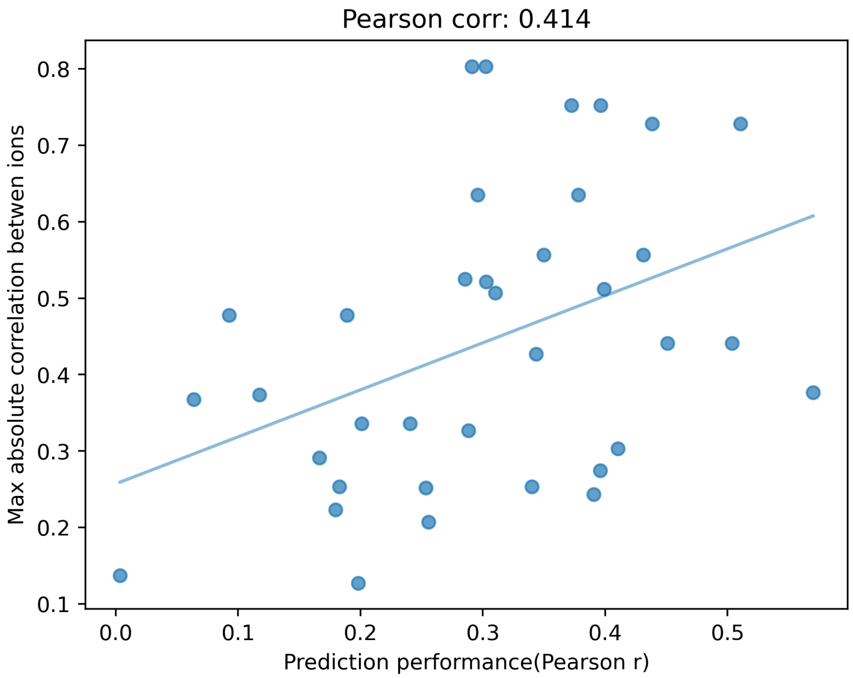

Figure S57: Scatter plot showing the relation between 1) the maximum absolute correlation between ionome values (y axis) and 2) the average prediction for each same ionome element (x axis).

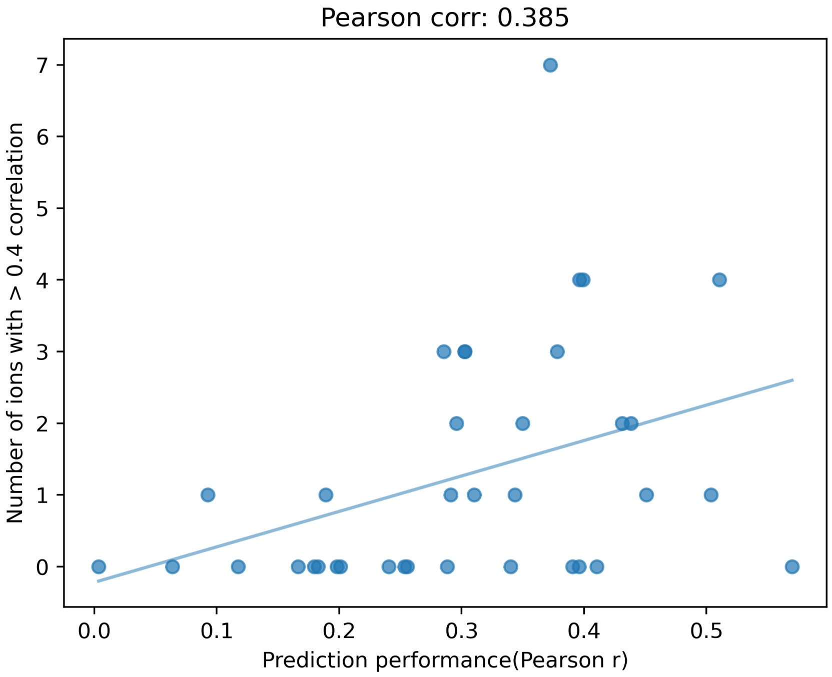

Figure S58: Scatter plot showing the relation between 1) the similarity of each element with the others and 2) the the average prediction for each element. In this case, the similarity between ionome elements is measured as the number of other elements that have a correlation greater than 0.4 with the target one.

References

[1] Ana Carolina AL Campos, William FA van Dijk, Priya Ramakrishna, Tom Giles, Pamela Korte, Alex Douglas, Pete Smith, and David E Salt. 1,135 ionomes reveal the global pattern of leaf and seed mineral nutrient and trace element diversity in arabidopsis thaliana. *The Plant Journal*, 106(2):536–554, 2021.
